# Odorant Receptors of the New Zealand Endemic Leafroller Moth Species *Planotortrix octo* and *P*. *excessana*

**DOI:** 10.1371/journal.pone.0152147

**Published:** 2016-03-22

**Authors:** Bernd Steinwender, Amali H. Thrimawithana, Ross Crowhurst, Richard D. Newcomb

**Affiliations:** 1 The New Zealand Institute for Plant & Food Research Limited, Auckland, New Zealand; 2 School of Biological Sciences, University of Auckland, Auckland, New Zealand; 3 Allan Wilson Centre for Molecular Ecology and Evolution, Auckland, New Zealand; USDA-ARS, UNITED STATES

## Abstract

Moths use their sense of smell to find food sources, mating partners and oviposition sites. For this they possess a family of odorant receptors (ORs). Some ORs are used by both sexes whereas others have sex-specific roles. For example, male moths possess ORs specifically tuned to sex pheromones produced by conspecific females. Here we identify sets of ORs from the antennae of New Zealand endemic leafroller moths *Planotortrix octo* (48 ORs) and *P*. *excessana* (47 ORs) using an RNA-Seq approach. Two orthologous ORs show male-biased expression in the adult antennae of both species (OR7 and OR30) and one other OR in each species was female-biased in its expression (PoctOR25, PexcOR14) by qPCR. PAML analysis conducted on male-biased ORs indicated positive selection acting on the male-biased OR7. The fact that OR7 is likely under positive selection, that it is male-biased in its expression and that its orthologue in *C*. *obliquana*, CoblOR7, responds to sex pheromone components also utilised by *Planotortrix* species, suggests that this receptor may also be important in sex pheromone reception in *Planotortrix* species.

## Introduction

In the insect world chemical cues play an important role in orientation and communication. Moths, for example, use their sense of smell to locate food, oviposition sites and mates [1,2]. For this they use an array of odorant receptors (ORs) located in sensilla that are found mainly on the insect’s antennae. Male moths use specialised ORs to locate conspecific females by tracking sex pheromones produced by specialised glands in the terminal segment of the female abdomen [3]. Females typically emit even numbered carbon chain fatty-acid derivates of 10–18 carbons in length, containing one or two double bonds along the fatty acid backbone [4]. For males, it is important to be able to discriminate between compounds produced by conspecific or hetero-specific females because mating mistakes are costly in terms of reproduction and can be fatal for both individuals [2].

New mating systems can only evolve if both female sex pheromone production (sender) and male reception (receiver) change. In females, changes in enzymes such as desaturases or fatty acid reductases can lead to the production of novel pheromone blends [5,6,7,8,9]. Even though we are beginning to understand the components involved in the female directed aspect of this ‘sender-receiver’ system and how they change, the evolution of the receiver has been less intensively investigated. To understand the evolution of new mating systems, we also need to consider sex pheromone perception and what changes underpin the evolution of this male-biased behaviour to promonte new mating systems and therefore speciation [10]. One hypothesis in this regard is the “asymmetric tracking hypothesis”. It suggests that if females are the limiting factor within a population, signal and response are less likely to be genetically linked, providing the opportunity for pheromone perception by the male to be more variable, with some males likely to be able to detect females that produce novel pheromone blends [11]. Indeed in some species such rare males have been found [12]. Alterations in a multigene family of receptors and differences in the structure within this family seem plausible for encoding the changes in male preference [13,14,15,16]. Insect ORs are ligand-gated ion channels consisting of seven transmembrane regions with an external C-terminus and an internal N-terminus [17,18,19]. The highly conserved odorant receptor co-receptor (Orco) [20], together with a ligand-binding OR, forms the active receptor complex. Ligand-binding ORs are more variable, especially in the N-terminal region, whereas the C-terminus is more conserved in its amino acid sequence [21].

ORs that bind sex pheromone components are located in specialised sensilla on the antennae called sensilla trichodea [22,23]. These long hair-like sensilla are more abundant in males than in females [24,25,26,27,28,29] and therefore receptors expressed in these sensilla typically show higher expression in male than in female antennae. These receptors also cluster phylogenetically in the “so called” sex pheromone receptor clade. However, in the light-brown apple moth, *Epiphyas postvittana*, and the New Zealand brown-headed leafroller moths, *Ctenopseustis obliquana* and *C*. *herana*, additional male-biased ORs were identified that do not fall into the sex pheromone receptor clade, although as yet there is no evidence that they are capable of responding to sex pheromone components [30,31].

Species of the endemic New Zealand brown-headed leafroller, *Ctenopseustis* (5 species), and green-headed leafroller, *Planotortrix* (7 species), are found throughout New Zealand with some species occurring all over the country, whereas others are restricted to specific regions [32,33] and preferences for host-plants range from specialists to highly polyphagous [34]. The most recent common ancestor for the two sister genera *Ctenopseusis* and *Planotortrix* is thought to have existed about 5 million years ago [35]. Some species within the genus *Ctenopseustis* have an estimated most recent common ancestor of less than 500 000 years ago with some sibling species within *Planotortrix* thought to have arisen even more recently [35]. Together with the Australian light-brown apple moth, *E*. *postvittana*, the polyphagous species *C*. *obliquana*, *C*. *herana*, *P*. *octo* and *P*. *excessana* form a complex of pest species in New Zealand [36] that have been investigated in some detail [33,36,37].

Females of *Ctenopseustis* and *Planotortrix* species produce compound blends consisting of unsaturated tetradecenyl acetates to attract conspecific males [38]. Typically double bonds are found at the 5, 7, 8 or 9 positions of the fatty acid-backbone of the molecule [38,39]. While the compounds differ in the position of the double bond in the fatty-acid backbone, they are always in the Z-isomer configuration [39]. Females in the four polyphagous species use different combinations of compounds in their sex pheromone blends to attract conspecific males. *Ctenopseustis obliquana* uses (*Z*)-5-tetradecenyl acetate (Z5-14:OAc) and (*Z*)-8-tetradecenyl acetate (Z8-14:OAc) in a ratio of 20:80, *C*. *herana* uses solely Z5-14:OAc, *P*. *octo* uses Z8-14:OAc and (*Z*)-10-tetradecenyl acetate (Z10-14:OAc) in a ration of 98:2 as a pheromone, and *P*. *excessana* females produce a blend of Z5-14:OAc and (*Z*)-7-tetradecenyl acetate (Z7-14:OAc) in a ratio of 60:40 [32]. The differential expression of desaturase genes is responsible for producing the different pheromone components [8,40]. The Δ10-desaturase gene in *Planotortrix* is thought to be differentially regulated by a *trans*-acting repressor and a *cis*-regulatory mutation in an activator binding site within the desaturase’s promoter region [40].

In addition to research conducted on female sex pheromone production in New Zealand endemic leafroller moths, sex pheromone reception has also been investigated. A study focusing on male sex pheromone perception in the genus *Ctenopseustis* showed in electrophysiological experiments that males of *C*. *herana* can detect the component Z8-14:OAc despite it not being a pheromone component in this species, however, it is used by the sibling species *C*. *obliquana* with a substantially different neurological response to the component [41]. Males of *C*. *obliquana* possess a large-spike amplitude olfactory sensory neuron that responds strongly to Z8-14:OAc, whereas *C*. *herana* males have a small-spike amplitude neuron that responds weakly to this component. Fluorescent calcium influx assays performed in HEK293 cells showed that the male-biased receptor OR7 in *C*. *obliquana* (CoblOR7) responds only to Z8-14:OAc, but its orthologue in *C*. *herana* (CherOR7) responds to Z7-14:OAc in addition to Z8-14:OAc [30]. Receptor *OR7* clusters phylogenetically in the sex pheromone receptor clade and investigations of both orthologs indicate that CoblOR7 is under positive selection, presumably because it is important in *C*. *obliquana* to have a receptor that is highly specific to the sex pheromone component Z8-14:OAc. Considering that *C*. *herana* uses Z5-14:OAc alone as its sex pheromone, *OR7* in this species seems to be under relaxed constraint [30]. Furthermore, the identification of ORs in *C*. *obliquana* and *C*. *herana* using transcriptome databases from male and female antennae as well as quantitative real-time PCR (qPCR) revealed high levels of similarity between orthologuous OR genes and male-biased expression of the same two genes, OR7 and OR30, in both sibling species.

Here we apply transcriptomic and bioinformatic tools to identify ORs in the New Zealand green-headed leafroller species, *P*. *octo* and *P*. *excessana*. We identify candidate sex pheromone receptors through examining expression differences of OR genes between the male and female antennae and conduct dN/dS analysis on male-biased receptors to test for evidence of selection acting on these genes.

## Material and Methods

### Insects

All insects were reared in the Plant & Food Research insect rearing facility at the Mt Albert Research Centre, Auckland, New Zealand. The *P*. *octo* colony originated from collections made in Canterbury in 1982 and the *P*. *excessana* colony was established from moths collected in Dunedin in 1998. Eggs were collected and kept in a humid environment. Larvae were reared individually in small glass tubes and fed on a general purpose diet [42] at 18°C. Pupae and adults were kept on a 16:8 light cycle at 20°C. Adult moths were provided with water via a soaked cotton cloth.

### Nucleic acid isolation

RNA isolation for transcriptome sequencing was executed from batches of 100 male and female antennae each dissected from 2–3 days old adult moths. RNA was extracted and purified using TRIzol (Life Technologies, Carlsbad, CA, USA) and total RNA was DNase treated using the TURBO DNA-free Kit (Life technologies) as described in [30].

RNA for qPCR experiments was also isolated from antennae dissected from 2–3 day-old adults, as well as whole bodies, using TRIzol following the manufacturer’s instructions. The initial screening for expression differences of ORs in male and female antennae was conducted with RNA extracted from ten pairs of antennae with subsequent qPCR experiments conducted on RNA extracted from single pairs of antennae. RNA was treated with DNase (DNaseI amplification grade, Invitrogen) following the manufacturer’s instructions and cDNA was synthesised using the iScript cDNA Synthesis Kit (Bio-Rad) as described in [30].

### Next-generation sequencing and bioinformatics

RNA-Seq libraries were constructed from both male and female adult antennae of *P*. *octo* and *P*. *excessana* using Illumina’s HiSeq 2000 standard protocols and sequenced at Macrogen (Seoul, South Korea). Quality score analysis on the read pairs for each library was undertaken using FastQC. In-house Perl scripts were used to trim all reads by 13 bases at their 5' ends and remove any read pairs containing Ns and mononucleotides. Mitochondrial contamination was removed by mapping RNA-Seq read pairs to a reference mitochondrial genome of *P*. *octo* assembled from a draft genome. Mapping was performed using bowtie (version 1.0.0) [43], with the reads mapping to the mitochondrial genome being removed. Read pairs were trimmed to a minimum quality threshold of 20 using fastq-mcf from the ea-utils package [44]. Duplicates within the read files for *P*. *octo* were removed using in-house Perl scripts prior to assembly, while the redundancy in the *P*. *excessana* sequences was removed after the assembly using cd-hit [45]. *De novo* assembly of the processed reads was performed for each of the individual libraries (S1 Table) with trans-ABySS (version 1.3.2) [46], where a k-mer series of 31 to 75 with an increment of two bases was used for the libraries of *P*. *octo*, whereas k-mer values from 31 to 71 were used for the *P*. *excessana* libraries.

To identify candidate ORs, tblastn [47] was used with amino acid sequences of ORs from the light-brown apple moth *Epiphyas postvittana* [48], as well as the ORs from each of the brown-headed leafroller moth species *Ctenopseustis obliquana* and *C*. *herana* [30], as queries. Full length sequences of ORs were obtained using tblastn against the transcriptomes of male and female antennae from *P*. *octo* and *P*. *excessana*. In some cases, a draft genome of *P*. *octo* (unpublished data) was used to extend sequences. The processed RNA-Seq reads were mapped onto a constructed set of OR sequences using bowtie (version 2.1.0) [49]. The resulting alignment was then used to obtain expected read counts using multiBamCov from the bedtools package (v2.16.2) [50] and cufflinks (v2.1.1) [51] for Fragments Per Kilobase of transcript per Million reads (FPKM).

Assembled OR sequence data were edited and aligned in Geneious 6.1.6 [52] using ClustalW with *C*. *obliquana*, *C*. *herana* and *E*. *postvittana* sequences. The model for phylogenetic trees was chosen by ModelTest [53] and maximum likelihood trees were generated in MEGA6 [54]. The dN and dS rates were estimated using codon-based substitution models in PAML version 4.7 [55] by using M0 as the model with one fixed ω ratio and the model M3, which has three categories of site with a free ω ratio for each site class [55]. Transmembrane domains were predicted using SPLIT 4.0 [56] at the transmembrane prediction server (http://split4.pmfst.hr/split/4/). The topology diagram was constructed using TOPO2 Transmembrane Protein Display [57] by the server at UCSF (http://www.sacs.ucsf.edu/TOPO2).

### Quantitative Real-Time PCR

Expression levels of ORs in male and female antennae and bodies were determined relative to α-tubulin, β-actin and elongation factor 1α [58]. Primers were designed to receptors found in *P*. *octo* and tested on the orthologous receptors from the sibling species *P*. *excessana* (S1 Table). Experiments were executed as described previously with three biological replicates and three technical replicates per biological replicate [30] using 20 ng of cDNA in a final reaction volume of 10 μl over 45 cycles using a LightCycler480 Real-Time instrument (Roche Diagnostics, Basel, Switzerland). The data analysis was also conducted as described previously [30], with the determination of the amplification efficiency and the cycle threshold values for each reaction conducted using the software LinRegPCRv11 [59] and expression levels were calculated as described in [48] using a modified version of the ΔCp method [40,60,61].

## Results

### Antennal transcriptomics and the OR genes

**Fig 1:**
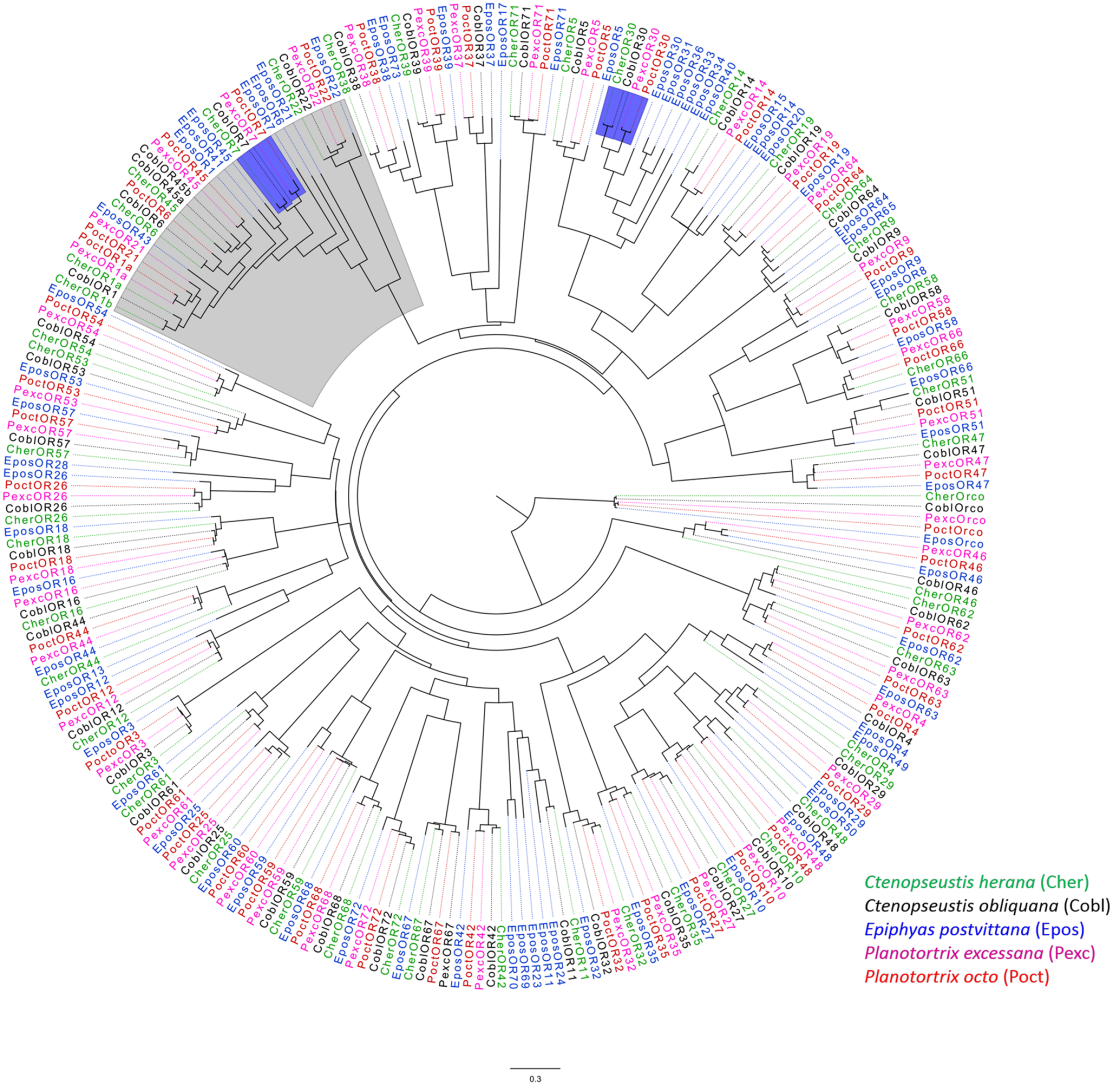
Phylogenetic analyses of odorant receptors from *Planotortrix octo*, *P*. *excessana Ctenopseustis obliquana*, *C*. *herana* and *Epiphyas postvittana*. Maximum likelihood rooted phylogeny of all odorant receptors from the five species. The tree is rooted with the odorant receptor co-receptor (Orco) The sex pheromone receptor clade is highlighted in grey, OR07 and OR30 (male biased) are highlighted in blue.

In total four RNA-Seq libraries were generated from the antennae of 2–3 day old adult male and female *P*. *octo* and *P*. *excessana* generating from 56,971,439 to 61,132,402 raw sequence reads per library (S2 Table). RNA-Seq data have been deposited into a Sequence Read Archive (SRA) database online (http://www.ncbi.nlm.nih.gob/) under the Accession Numbers 243920 and 243922. Assemblies from RNA-Seq data ranged in their number of contigs from 125,512 to 228,676 (S3 Table). Forty-eight OR genes from *P*. *octo* and 47 from *P*. *excessana* were identified through blast analysis and corresponding protein sequences were derived. Except for PexcOR16, all ORs from outside the sex pheromone receptor clade were represented by orthologous sets in both *Planotortrix* species (Fig 1). A comparison with ORs identified in species of the sister genus *Ctenopseustis*, *C*. *obliquana* and *C*. *herana* [30], revealed that OR60 is exclusively expressed in antennae of *P*. *octo* and *P*. *excessana* and OR11 is found in *C*. *obliquana* and *C*. *herana* antennae only. Orthologous ORs within *Planotortrix* share levels of amino acid identity from 87.7% to 100% (S4 Table). The clade containing sex pheromone receptors from other moth species including *C*. *obliquana* and *C*. *herana* is supported by bootstrap analysis (81% from 1000 bootstrap replicates). Within the sex pheromone receptor clade all identified ORs are present as orthologues in all four New Zealand endemic leafroller species apart from OR6 that was not found among the *P*. *excessana* transcripts (Fig 1).

### Gene expression of ORs

Sex pheromone receptors are in general more highly expressed in male antennae than in female antennae [28,62], therefore we sought to identify receptors with male-biased expression in adult antennae (S1 and S2 Figs). Using a two-fold cut off in FPKM scores two OR genes in each of *P*. *octo* and *P*. *excessana* were identified as male-biased, OR7 and OR30. Orthologues of these ORs are also male-biased in *C*. *obliquana* and *C*. *herana* with OR7 falling into the sex pheromone receptor clade and OR30 postitioned outside of this clade (Fig 1). RNA-Seq counting, with a minimum count of 10,000 FPKM and a two-fold cut off also revealed ten female-biased ORs in *P*. *octo* (PoctOR10, PoctOR14, PoctOR22, PoctOR25, PoctOR27, PoctOR39, PoctOR47, PoctOR64, PoctOR66 and PoctOR71) and nine in *P*. *excessana* (PexcOR14, PexcOR22, PexcOR25, PexcOR26, PexcOR39, PexcOR57, PexcOR64 and PexcOR66). Because the analysis using FPKM values is not very stringent, we verified sex-biased expression by gene expression experiments (qPCR).

**Fig 2:**
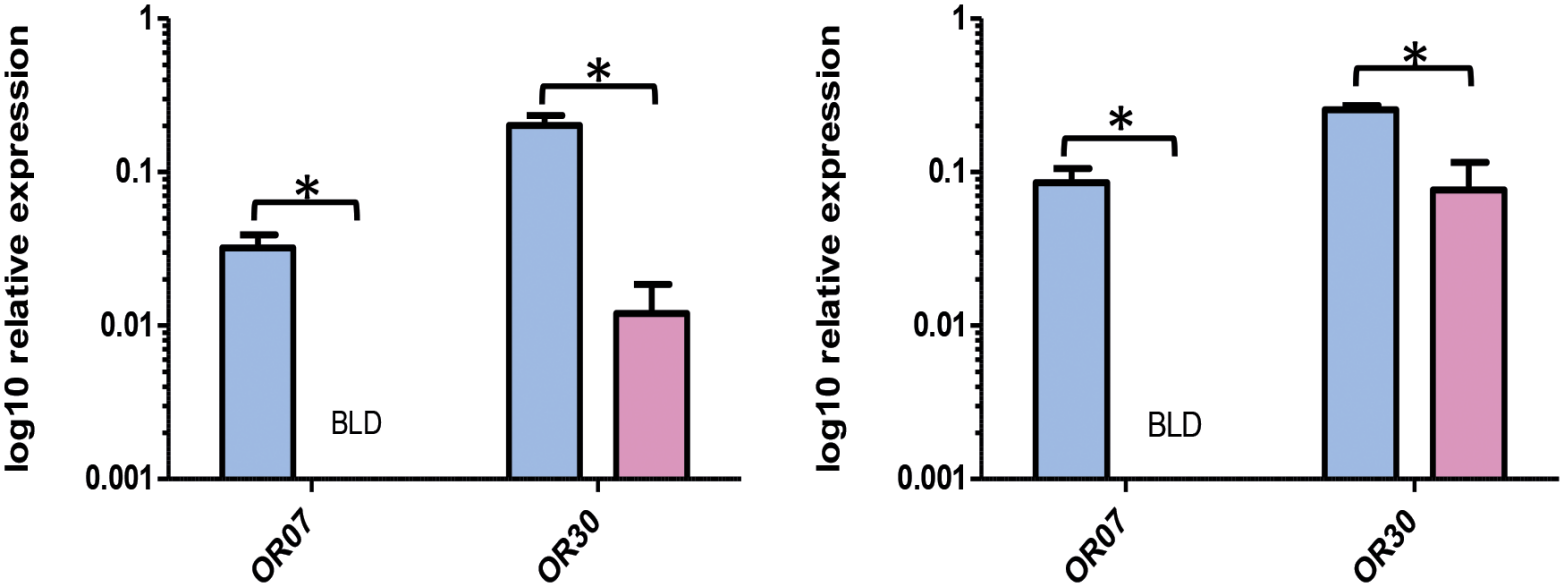
Relative expression of the odorant receptors OR7 and OR30 in male (blue) and female (red) antennae of adult. *Planotortrix octo* (left) and *P*. *excessana* (right). CT values were normalised to the housekeeping genes α-tubulin, β-actin and elongation factor 1α. BLD = below limits of detection.

Twenty-five OR genes in *P*. *octo* and twenty-six in *P*. *excessana* were investigated for their levels of gene expression using qPCR (S3 and S4 Figs). Among the investigated genes were the male-biased receptors from the FPKM analysis, OR7 from the pheromone receptor clade, as well as OR30. Both genes were also male-biased by qPCR in both species (Fig 2; PoctoOR7 *P* = 0.014; PexcOR7 *P* = 0.009; PoctOR30 *P* = 0.014; PexcOR30 *P* = 0.004). In addition to the male-biased receptors, female-biased receptors were also identified by qPCR. In *P*. *octo*, one receptor was confirmed as being more highly expressed in female than male antennae (PoctOR25, *P* = 0.002), but not PoctOR10 (*P* = 0.142), PoctOR14 (*P* = 0.246), PoctOR22 (*P* = 0.859), PoctOR27 (*P* = 0.743), PoctOR39 (*P* = 0.382), PoctOR47 (*P* = 0.657) and one OR from *P*. *excessana* (PexcOR14, *P* = 0.020) but not PexcOR22 (*P* = 0.552), PexcOR25 (*P* = 0.564), PexcOR26 (*P* = 0.473), PexcOR32 (*P* = 0.211) and PexcOR39 (*P* = 0.126). No expression differences in any of the other ORs that were investigated were observed.

### Sequence comparison of ORs

**Fig 3:**
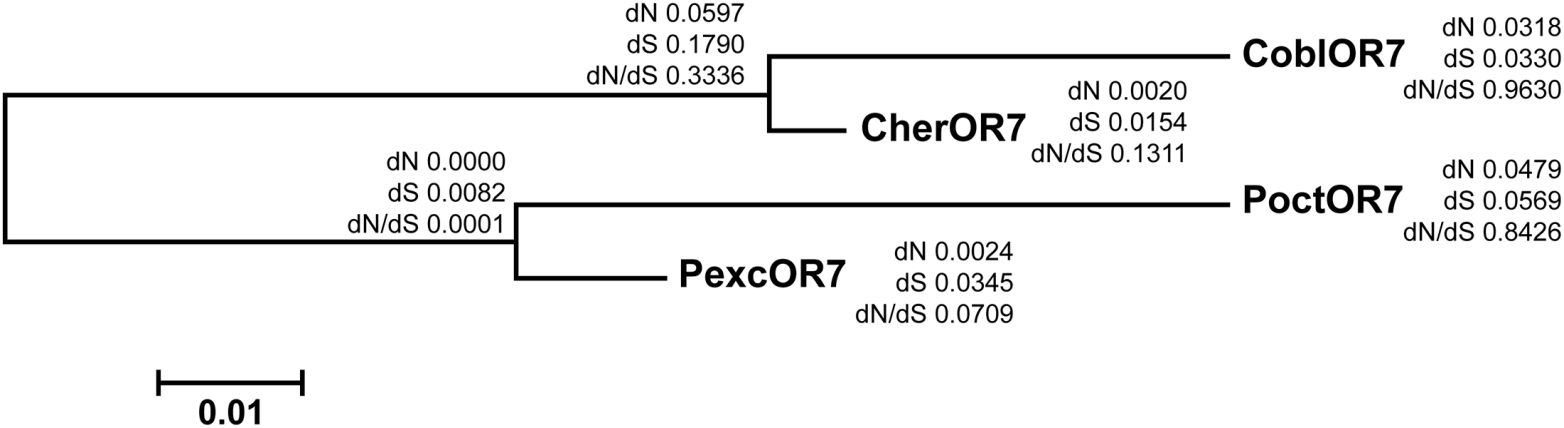
PAML analysis of OR7 across the endemic New Zealand leafroller moths *Ctenopseustis obliquana*, *C*. *herana*, *Planotortrix octo* and *P*. *excessana*. Maximum likelihood tree of OR7 othologues from *C*. *obliquana* (CoblOR7), *C*. *herana* (CherOR7), *P*. *octo* (PoctOR7) and *P*. *excessana* (PexcOR7). dN, dS and dN/dS values were generated using the M3 model.

Sequence comparison among orthologues of OR7 from all four sibling species revealed a dN/dS ratio of ω = 0.8426 for *P*. *octo* (43.8 non-synonymous and 18.8 synonymous changes) and ω = 0.0709 for *P*. *excessana* (2.2 non-synonymous and 11.4 synonymous changes) (Fig 3). A likelihood ratio test (LRT) of M0 (with one fixed ω ratio) vs M3 (with three categories of site with a free ω ratio for each site) indicated variability of the ω ratio at sites across the coding sequence of the male-biased receptor OR7 (*P* = 0.006). A comparison of the more stringent models M7 (beta neutral model) with M8 (beta plus ω), and M8 with M8a (M8 with a fixed ω at 1) were not significant.

**Fig 4:**
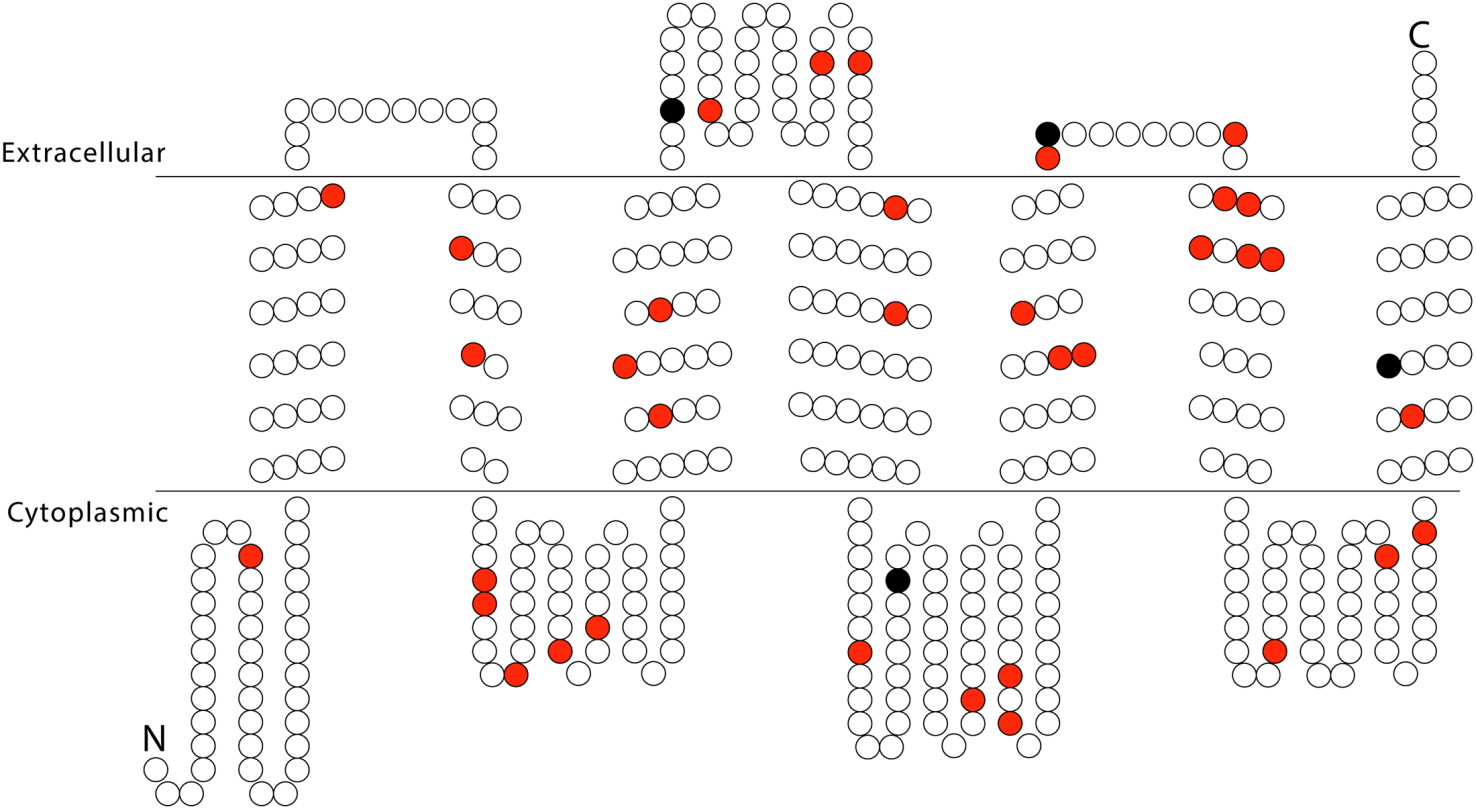
Predicted transmembrane topology of OR7. Variable sites between *P*. *octo* and *P*. *excessana* are highlighted. Red dots indicate the position of amino acid substitutions in *P*. *octo*, and black dots amino acid substitutions in *P*. *excessana* compared to a predicted common ancestor. The double line indicates the transmembrane region, with extracellular and cytoplasmic sides labelled.

There are 71 nucleotide differences between PoctOR7 and PexcOR7 comprising 26 synonymous and 45 non-synonymous substitutions at 39 amino acid positions (Fig 4). A test for equal distribution of non-synonymous substitutions across the fifteen regions (N terminal region, seven transmembrane regions, three intracellular loops, three extracellular loops, and the C terminal region) was not rejected (*X*^*2*^ = 14.84, *P* = 0.39).

**Fig 5:**
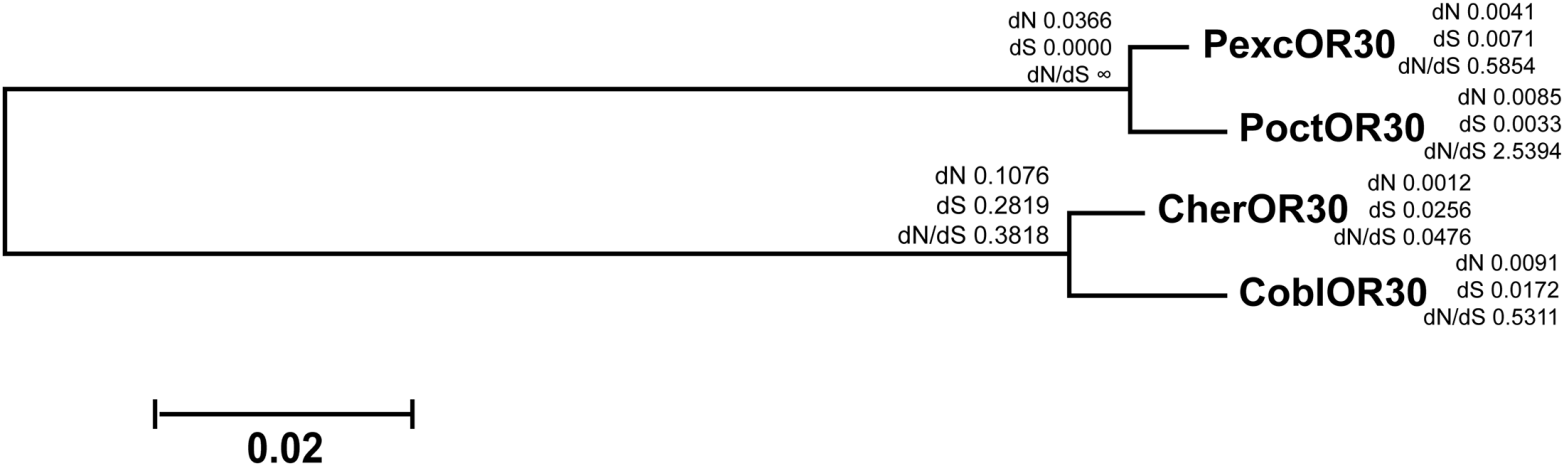
PAML analysis of OR30 across endemic New Zealand leafroller moths. Maximum likelihood tree of OR30 othologues from *Ctenopseustis obliquana* (CoblOR30), *C*. *herana* (CherOR30), *Planotortrix octo* (PoctOR30) and *P*. *excessana* (PexcOR30). dN, dS and dN/dS values were generated using the M3 model.

For the other male biased OR, OR30, the likelihood ratio comparing M0 vs M3 was not significant (*P* = 0.24; 2Δl = 8.0071). The dN/dS ratio for *P*. *octo* ω = 2.5394 (7.4 non-synonymous and 1.0 synonymous substitutions) and for *P*. *excessana* ω = 0.5854 (3.6 non-synonymous and 2.2 synonymous substitutions) (Fig 5).

**Fig 6:**
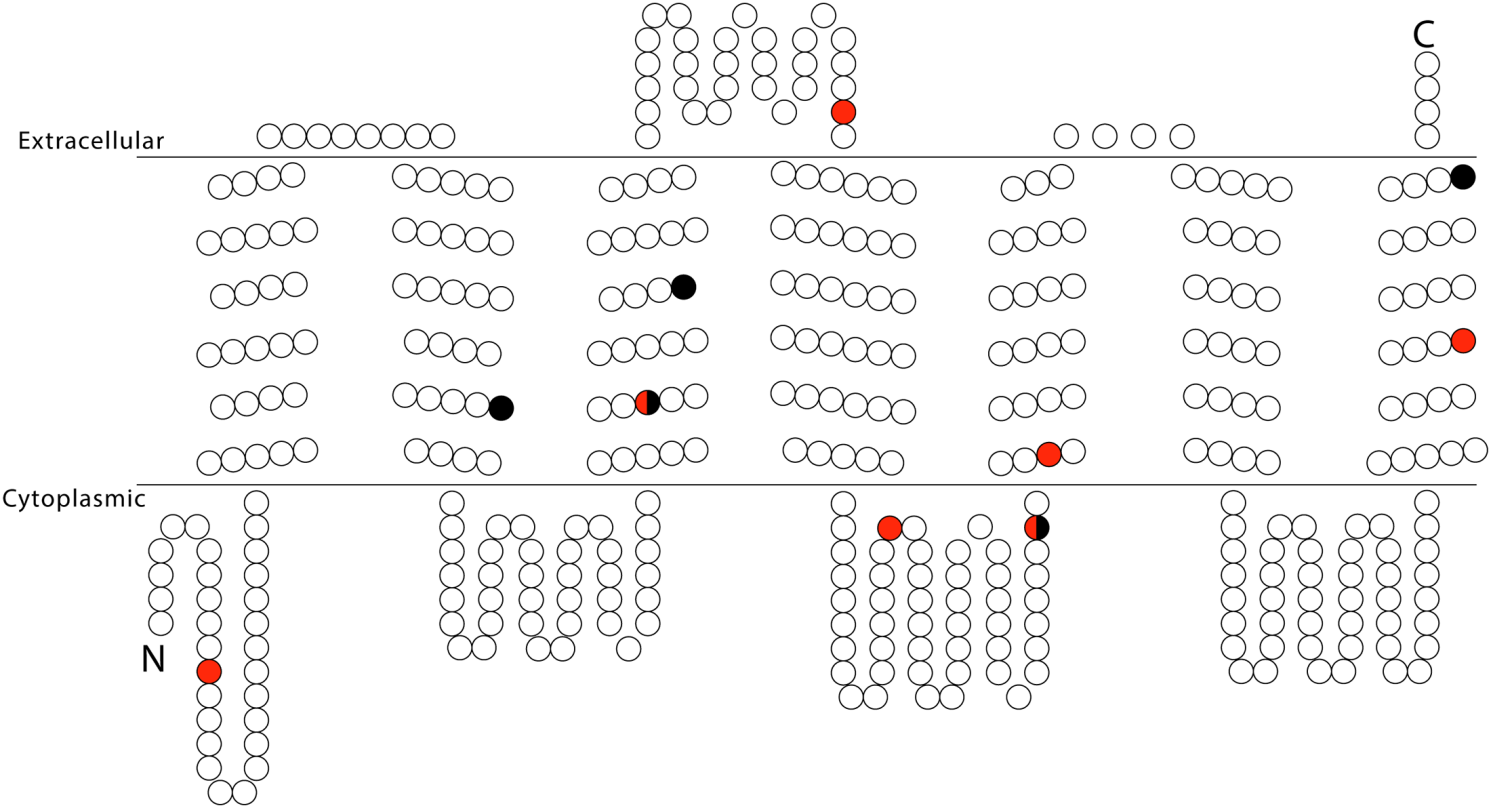
Predicted transmembrane topology of OR30 from *P*. *excessana* and *P*. *octo*. Variable sites highlighted in red indicating amino acid substitutions in *P*. *octo*, black indicating substitutions in *P*. *excessana*, red-black indicating independent substitutions in *P*. *octo* and *P*. *excessana* compared to a predicted common ancestor. The double line indicates the transmembrane region, with extracellular and cytoplasmic sides labelled.

We note that because the number of substitutions in OR30 is low, small changes in the ratio of synonymous to non-synonymous substitutions has a large impact on ω values. There are 14 nucleotide differences between PoctOR30 and PexcOR30 (Fig 6). No deviations from an equal distribution of non-synonymous substitutions among the fifteen regions of the receptor were revealed for OR30 from *P*. *octo or P*. *excessana*.

## Discussion

Using a transcriptomics approach we have identified 48 odorant receptors (ORs) from *P*. *octo* and 47 from *P*. *excessana*. This greatly expands the number of ORs identified from these species from the three previously found in each [63]. The number of ORs identified in the two *Planotortrix* species is comparable to the 47 ORs identified in species of the sister genus *Ctenopseustis* [30], but is less than the number identified in the related tortricid species *Epiphyas postvittana*, where 70 ORs were identified [48]. In another tortricid moth, *Cydia pomonella*, 43 ORs were identified [64]. It is possible that more ORs remain to be identified in these species. In addition to the ORs, the antennal transcriptomes can be used to identify other genes encoding proteins involved in the process of chemoreception such as other classes of receptors (ionotropic receptors, gustatory receptors), odorant/pheromone binding proteins and odorant degrading enzymes.

Odorant receptors associated with sex pheromone perception usually fall into the “so-called” sex pheromone receptor clade and show higher levels of expression in the antennae of males compared with females [26,65,66]. Five ORs from *P*. *excessana* and six from *P*. *octo* fall into the sex pheromone receptor clade. However, only OR7 showed significant male-biased expression in the two species.

Within the pheromone receptor clade no clear orthology between receptors from *E*. *postvittana* and the two *Planotortrix* species (Fig 1) can be found, compared with ORs outside the clade. This was also observed between ORs from *E*. *postvittana* and species within the genus *Ctenopseustis* [30]. However, between the genus *Planotortrix* and *Ctenopseustis*, even in the highly variable pheromone receptor clade orthologuous genes could be identified. This is despite the fact that ORs within the pheromone receptor clade seem to evolve faster than other ORs [63]. The presence of orthologues within the sex pheromone receptor clade across the *Planotortrix* and *Ctenopseustis* genera could indicate the conserved ability to detect pheromone components of a common ancestor since some pheromone components are used repeatedly across the two genera (eg. Z5-14:OAc and Z8-14:OAc).

All OR genes outside the pheromone receptor clade are present as highly conserved orthologous pairs in the two *Planotortrix* species, except for OR16, which was only found expressed in the antennae of adult *P*. *excessana*. Similarly, between the two genera *Planotortrix* and *Ctenopseustis* all OR genes form orthologous sets, except for OR60 that is present only in *Planotortrix* and OR11 that is present only in *Ctenopseustis*. Among these orthologous sets of genes is the odorant receptor co-receptor (Orco) and OR3 which have been described previously [63]. A high degree of similarity of OR genes in the four species of New Zealand native leafroller moths indicates that the function of these ORs is likely conserved. Sequence similarity in OR genes of closely related species has also been shown in two *Helicoverpa* species [67].

The male-biased receptors PoctOR7 in *P*. *octo* and PexcOR7 in *P*. *excessana* reside within the clade where pheromone receptors of several lepidopteran species are found [27,68,69,70]. PoctOR7 has a higher dN/dS value than PexcOR7. Though not statistically significant, a higher proportion of substitutions were observed in the sixth transmembrane region of PoctOR7 (Fig 4). This is also the case in the orthologous receptor CoblOR7 in *C*. *obliquana* where more substitutions were also found in this region [30]. In the European and the Asian corn borer a single mutation in the sequence of an OR gene changes the specificity of the receptor to (E)-11-tetradecenyl acetate [16]. The threonine to alanine substitution is found in the third transmembrane region (TM3) at amino acid position 148 [16]. Interestingly, *P*. *excessana* and *P*. *octo* also exhibit a difference in their amino acid sequence at the same position, a serine or proline, respectively. Similarly, *C*. *herana* and *C*. *obliquana* differ at this position with a serine or leucine present, respectively [30]. In the sibling species of both genera the amino acid in position 148 of the ancestral version of OR7 changes from a polar, hydrophobic amino acid (serine) to a non-polar, hydrophilic amino acid (leucine in *C*. *obliquana* and proline in *P*. *octo*). It is possible that this substitution in OR7 of the New Zealand endemic leaftroller species may encode for differences in ligand binding for this receptor among the species. The orthologues, CoblOR7 and CherOR7 have been tested for response to ligand in HEK293 cell-based calcium influx assays and were found to respond to the sex pheromone component (Z)-8-tetradecenyl acetate (Z8-14:OAc), which is used in *C*. *obliquana* as the major component of the sex pheromone blend [30]. CherOR7 responded to Z8-14:OAc and (Z)-7-tetradecenyl acetate (Z7-14:OAc) even though neither of these compounds is used as a component of the sex pheromone in this species. It was suggested that CherOR7 is under relaxed constraints giving it the opportunity to mutate without fitness consequences for *C*. *herana* males since Z8-14:OAc is not required in this species. PoctOR7 and PexcOR7 have also been tested for response to ligand in HEK293 cells but to date efforts to find an activating ligand for these two receptors have been unsuccessful. Female *P*. *octo* use Z8-14:OAc as a pheromone and *P*. *excessana* females produce Z7-14:OAc and (Z)-5-tetradecenyl acetate (Z5-14:OAc). Following the argument made in Steinwender et al. (2015) that the ancestral version of OR7 responded to Z7-14:OAc, positive selection may have acted on amino acid substitutions in PoctOR7 to render it more selective for Z8-14:OAc. In contrast, the very low numbers of non-synonymous substitutions in PexcOR7 compared to the predicted ancestral sequence may indicate the action of stabilising selection, since *P*. *excessana* uses Z7-14:OAc as a sex pheromone component. Results acquired from cell assays on CoblOR7 and CherOR7 [30] suggest an important role for OR7 as a pheromone receptor in New Zealand endemic leafroller moths.

The second receptor identified as male-biased in all four New Zealand endemic leafroller species is OR30. This receptor resides outside the pheromone receptor clade and has an orthologue in *E*. *postvittana*, which is also more highly expressed in male antennae [48]. Compared to OR7, OR30 shows fewer amino acid differences when comparing orthologues of the sibling species *C*. *obliquana/C*. *herana* and *P*. *octo/P*. *excessana* (Fig 6). One reason for this could be the higher rate of evolution of OR7, observed for receptors within the pheromone receptor clade [63]. Another explanation could be a conserved function for OR30. Even though no functional data are yet available for OR30 in either the New Zealand native leafrollers or *E*. *postvittana*, this OR could be a candidate for binding Z5-14:OAc in *Ctenopseustis* and *Planotortrix*. The compound Z5-14:OAc is used by *C*. *obliquana*, *C*. *herana* and *P*. *excessana* as a sex pheromone component and is also used in other species within the genera *Ctenopseustis* and *Planotortrix* [32,39,71,72,73,74,75,76].

One OR in *P*. *octo* (PoctOR25) and one in *P*. *excessana* (PexcOR14) were confirmed as being female-biased in their expression in antennae by qPCR (S3 and S4 Figs). Even though not significantly female-biased by qPCR in either of the two *Planotortrix* species, OR22 showed a trend towards higher expression in female than in male antennae by RNA-Seq-counting. In *C*. *obliquana*, *C*. *herana* and also in *E*. *postvittana*, OR22 was also detected as being female-biased [30,48]. In total, two sets of orthologous receptor genes were more highly expressed in female antennae than in males in all four species of New Zealand endemic leafroller moths by RNA-Seq counting (*OR22* and *OR25*; S1 and S2 Figs and [30]), suggesting an important role for these receptors in adult females. However, gene expression analysis by qPCR only confirmed a significantly higher expression in female than in male antennae for OR25 in *P*. *octo* and OR 14 in *P*. *excessana*. Further investigation of the female-biased receptors in these species within the endemic New Zealand leafroller complex will be useful to understand the evolution of host specificity and polyphagy [32]. Also, it has been shown in several moth species that males produce short range pheromones. These pheromones are used in close proximity and are an important factor during courtship [77,78,79,80,81]. Female-biased ORs in *Planotortrix* and *Ctenopseustis* species could be involved in detecting pheromones produced by males and facilitate mate recognition.

In conclusion, we provide a large resource for chemosensory genes from antennae of the sibling species *P*. *octo* and *P*. *excessana*. We have identified ORs, including candidates for sex pheromone receptors, with future functional work required to examine the role of these receptors.

## Supporting Information

S1 Fig RNA seq-count data (Fragments Per Kilobase of exon per Million fragments mapped) of all identified OR genes in *P*. *octo*. (PDF)

S2 Fig RNA seq-count data (Fragments Per Kilobase of exon per Million fragments mapped) of all identified OR genes in *P*. *excessana*. (PDF)

S3 Fig qPCR results for investigated *P*. *octo* ORs. Mean (±SEM) relative expression to the housekeeping genes α-tubulin, β-actin and elongation factor 1α (n = 3).
(PDF)

S4 Fig pPCR results for investigated *P*. *excessana* ORs. Mean (±SEM) relative expression to the housekeeping genes α-tubulin, β-actin and elongation factor 1α (n = 3).
(PDF)

S1 Table Primers for qPCR. (PDF)

S2 Table Summary of read data for transcriptome assemblies. (PDF)

S3 Table Summary of metrics for *P*. *excessana* and *P*. *octo* transcriptome assemblies. (PDF)

S4 Table Sequence similarities between orthologous odorant receptors of *P*. *octo* and *P*. *excessana*. (XLSX)

S5 Table Summary of genes identified in *P*. *octo* and *P*. *excessana*. (PDF)
