# Supplementary figures and images for: Odorant Receptors of the New Zealand Endemic Leafroller Moth Species Planotortrix octo and P. excessana
Source: PLoS One. 2016 Mar 22;11(3):e0152147. doi: 10.1371/journal.pone.0152147 (PMC4803216; doi:10.1371/journal.pone.0152147)

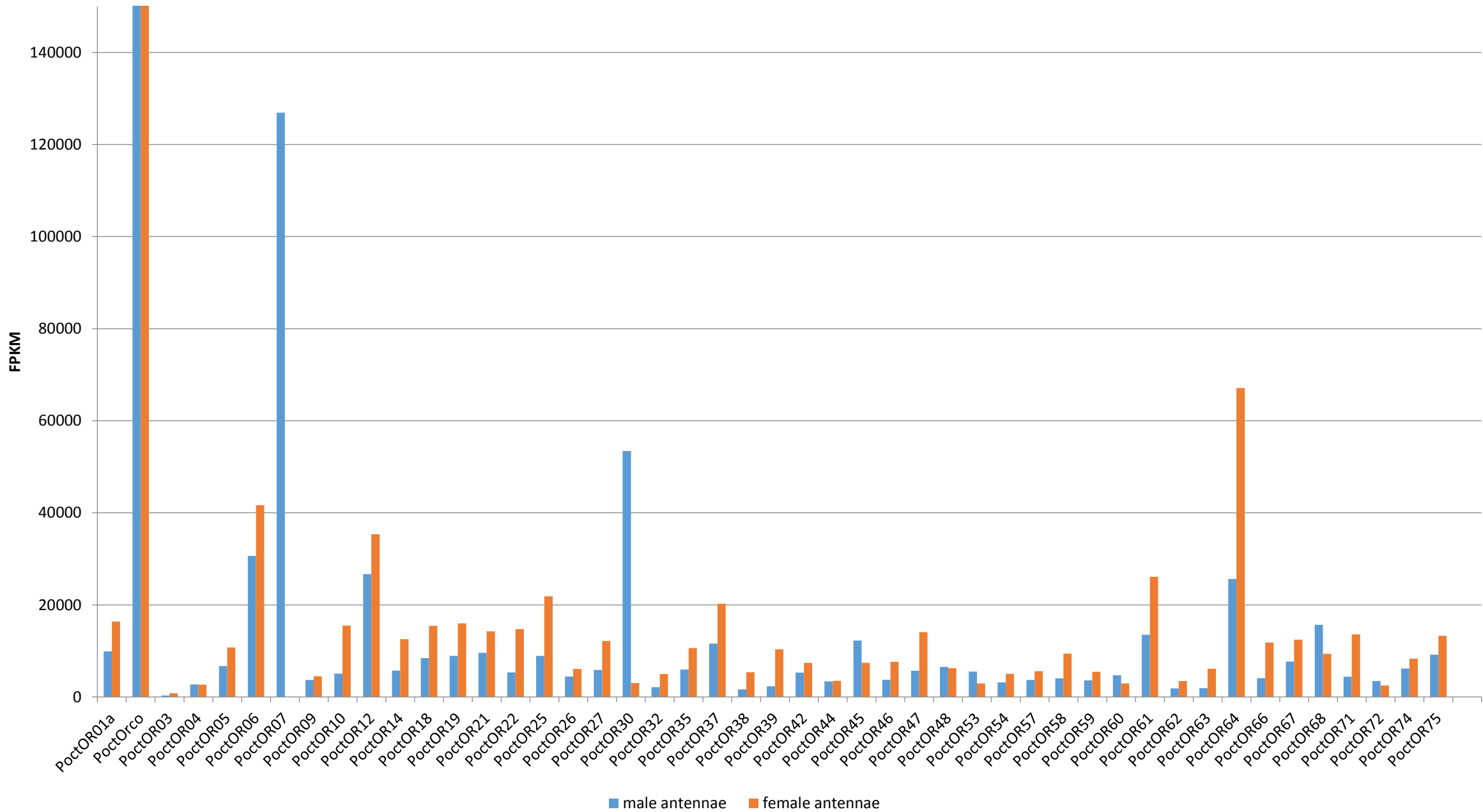

Supplement: S1 Fig — (PDF) [file pone.0152147.s001.pdf]

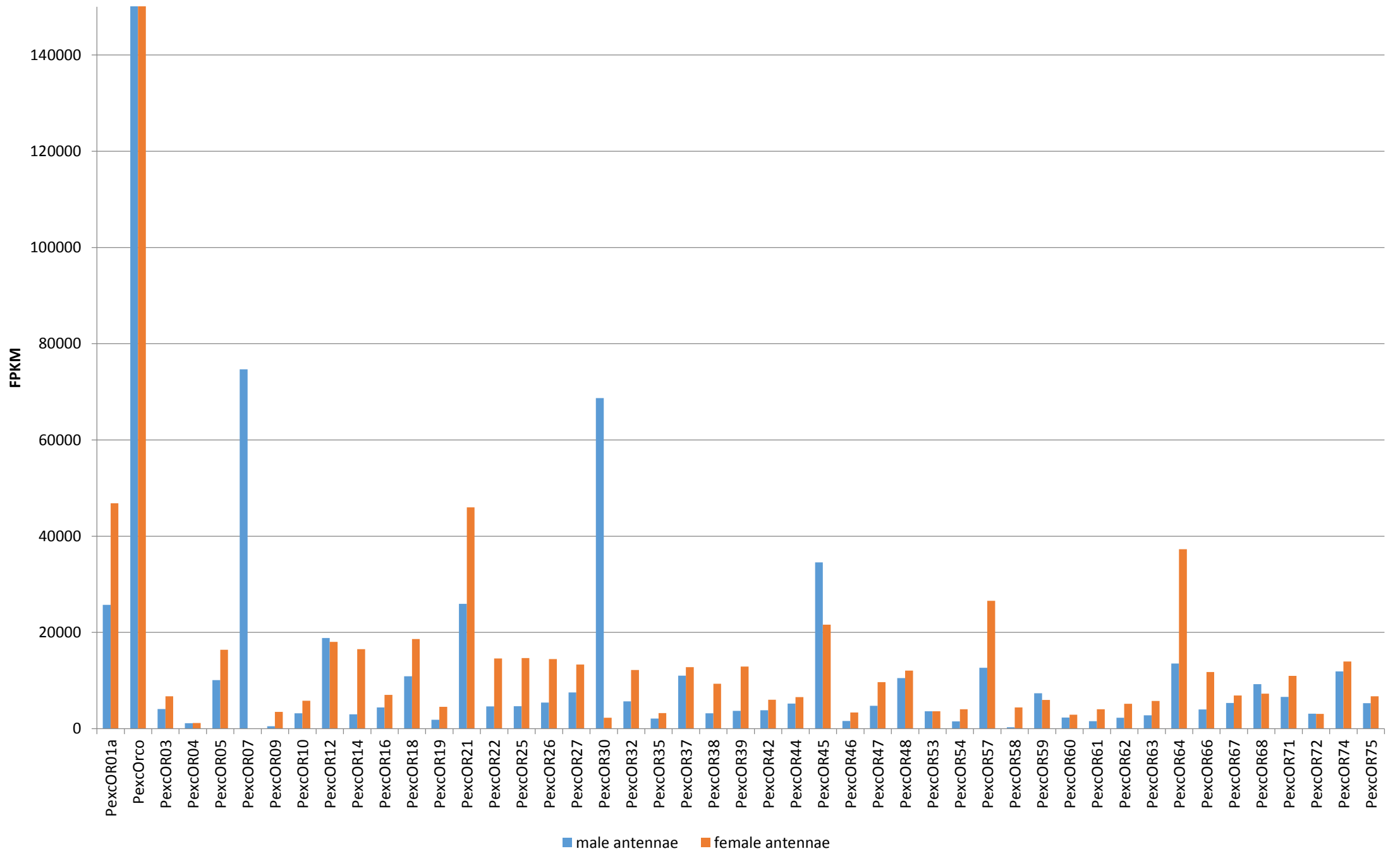

Supplement: S2 Fig — (PDF) [file pone.0152147.s002.pdf]

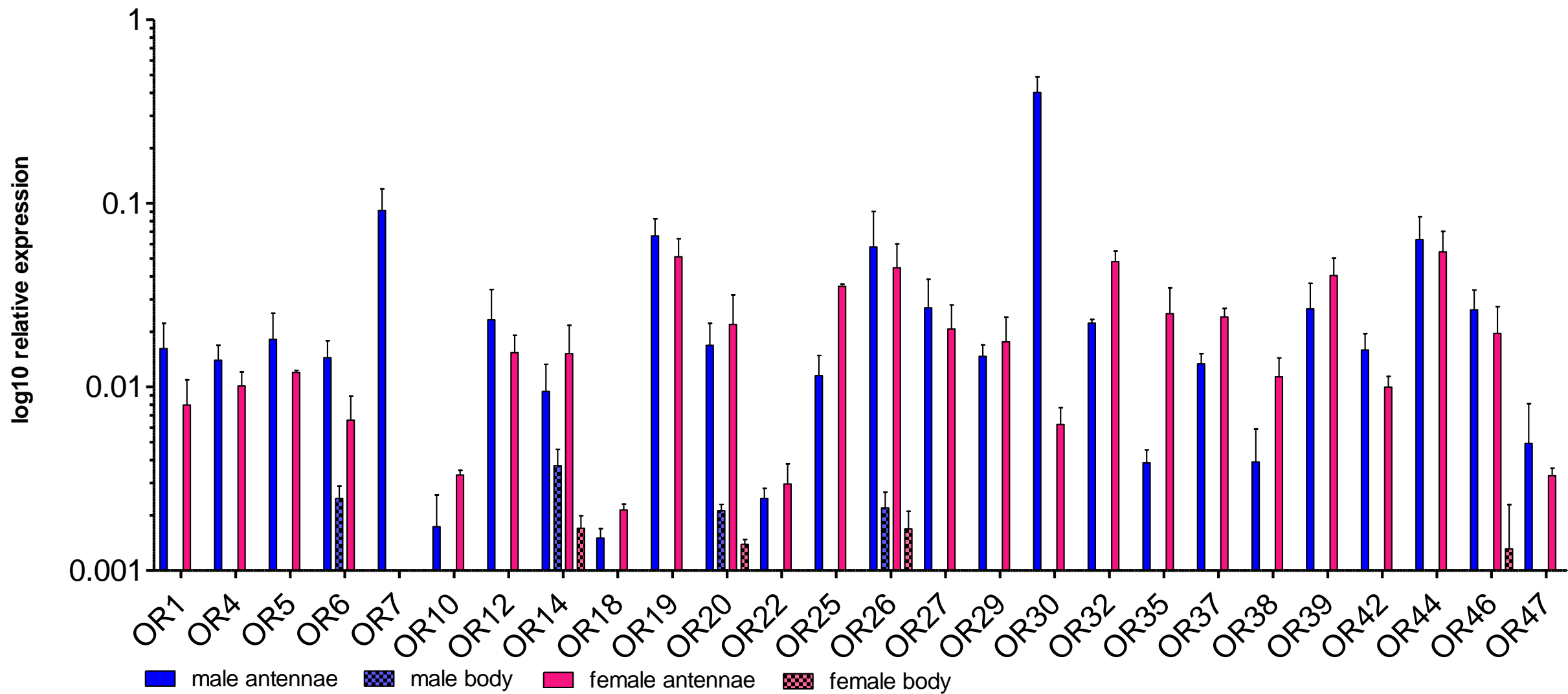

Supplement: S3 Fig — Mean (±SEM) relative expression to the housekeeping genes α-tubulin, β-actin and elongation factor 1α (n = 3). (PDF) [file pone.0152147.s003.pdf]

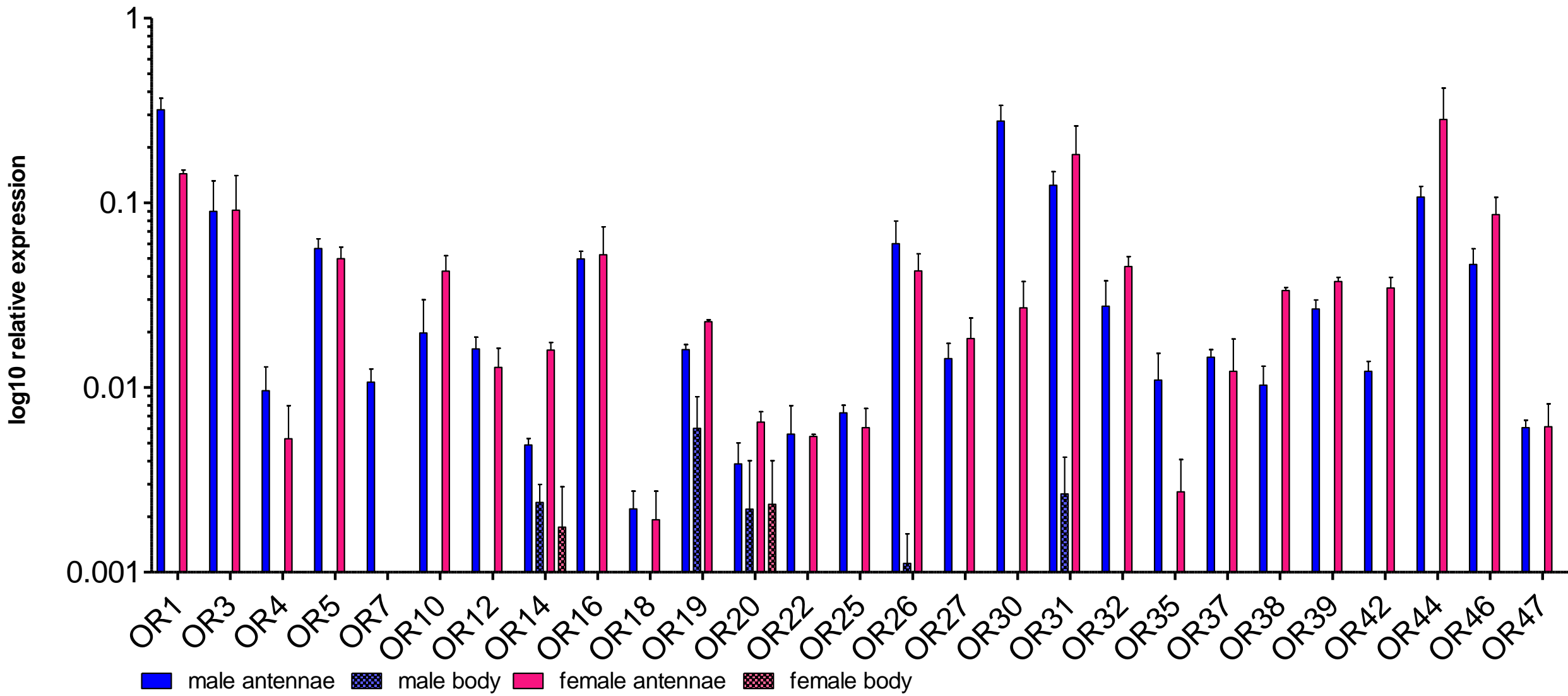

Supplement: S4 Fig — Mean (±SEM) relative expression to the housekeeping genes α-tubulin, β-actin and elongation factor 1α (n = 3). (PDF) [file pone.0152147.s004.pdf]
