# Supplementary material for: Odorant Receptors of the New Zealand Endemic Leafroller Moth Species Planotortrix octo and P. excessana
Source: PLoS One. 2016 Mar 22;11(3):e0152147. doi: 10.1371/journal.pone.0152147 (PMC4803216; doi:10.1371/journal.pone.0152147)
Supplement: S1 Table — (PDF) [file pone.0152147.s005.pdf]

Gene	Sequence forward primer (5’-3’)	Sequence reverse primer (5’-3’)
**OR01**	AGTCGATGCAGTGTACGGTTTGCC	GCAGGCTTTCAGCGCCAACG
**Orco**	ACGCAGCAAGACTTCGGTAT	TACGTGTTTATGGCGTTCCA
**OR03**	CAGACCATCAGGCAGCTGAGAATCC	CAATGCTGCTGCCAGTTCTCCC
**OR04**	TGCCACTGAGTACGTGGACATTTGG	ATGCTGACCCAGTCCTTTTGGAACG
**OR05**	CCGTTACTGACGACGTGGACACC	AGCCTCCAGCCACTTGTCTTGC
**OR06**	ATTGCGGAGATAAGACATCCGTCCC	AGTGGGCGTAGCACGGTAGG
**OR07**	CTGGAATAATGGCATGTTCG	GCAGAAGACGCAGGATAGGT
**OR10**	GTGTTCAGTCAAATTGTCGAGCTCTGG	CGATTGCCACAGGACGTAAGTAATGG
**OR12**	ACTTTACCGAAATTGCAGTGACCACC	ATTGTGTGTTTCCACGATTTTCCTGCC
**OR14**	GCAGAATGGTCGCTTTCAT	TCACAGACACGAAAAGTTCCA
**OR16**	AGCACTCTCCGCAGTACGAGC	TGAGCGCAGCCGAAGATGACC
**OR18**	TTGCGCGGGATAGACCGTGG	CGACTCTTGAAAACGCAACAAAATCGC
**OR19**	TGTTGAAAGGCAATGAGATGGAGGAGG	ACGTACACAGACTGACCGAGTTTGC
**OR20**	TCAACCGCGCCGAAGTCTACC	AACGTCAGCACGCAAAAGCCC
**OR22**	CATGGACTGGTTGCTATGGA	TGGGTAATAAGCCAGGTTCG
**OR25**	CCTTGCGTCTGGAGAGGTAT	TCTTTAAATTGCGCCCTCAT
**OR26**	ATGGAATCCGCATATTGCATGGATTGG	GGCACAATCTTGCCAGCCTTTGG
**OR27**	CAGGCACTCCAGAATGTTCA	GCACAAATCATCAAGGAGCA
**OR29**	TCACACGGCAAGACGTGAGGG	GCTGAGGCCGCTGTTTGGC
**OR30**	TGTTGTGGTCTGGGTGGTCATAACG	CTTCCTCGGGCAAGGAGTCGG
**OR32**	TGAACGCTACACAAGAAAACACACGG	CAGCGCGTATTCGACGAACGG
**OR35**	ATGTGGTTGCCTGGGATACT	ACCATGGAATCGATGACACA
**OR37**	TCTCCTGGGTCAGAGGCTTA	ACTGGTTTCTGAGCCTGCAT
**OR38**	CTCACGGTCTACGCTCCCGC	GGTAGCCGTTGGCAATGATTTTGGG
**OR39**	CGAAGCCGCTGTGTACGATTGC	CGCCCGCTGACAGAATCAAGG
**OR42**	CGAGAGTACGAAAGTCAGTGATTCGGC	ACTTATTCGCAGTCAGCCGTTGTGG
**OR43**	CCTACGAGTCGACATGGACA	AACCTGGCTGTAAGTCTGCAT
**OR44**	ACGTGTCGCTGATCGAGAGTGC	TCCCAACCAAACAGAGGACCATAACG
**OR46**	TGAGCTGTGACCGCGAGACG	TCGTAGGGATACCAAGCTCTCAAAGGC
**OR47**	AGTATGGGTTGCAGGTTTCG	TATCTTGGATGGCTCGGTTT
**OR52**	AATACTCCATCGTGACGGAAC	GTCTGTCAACCCTCTTAGCTTGA
