# Supplementary material for: Odorant Receptors of the New Zealand Endemic Leafroller Moth Species Planotortrix octo and P. excessana
Source: PLoS One. 2016 Mar 22;11(3):e0152147. doi: 10.1371/journal.pone.0152147 (PMC4803216; doi:10.1371/journal.pone.0152147)
Supplement: S2 Table — (PDF) [file pone.0152147.s006.pdf]

**Species**	**Library**	**Read length (bp)**	**Raw read pairs**	**Read pairs after trimming**
P.excessana	Female antennae	101	61132402	49676295
	Male antennae	101	69668522	61224161
P.octo	Female antennae*	101	56971439	5302448*
	Male antennae*	101	61533754	6227107*

* The library underwent removal of duplicates prior to assembly
