# Supplementary material for: Odorant Receptors of the New Zealand Endemic Leafroller Moth Species Planotortrix octo and P. excessana
Source: PLoS One. 2016 Mar 22;11(3):e0152147. doi: 10.1371/journal.pone.0152147 (PMC4803216; doi:10.1371/journal.pone.0152147)
Supplement: S3 Table — (PDF) [file pone.0152147.s007.pdf]

	P.excessana		*P.octo*	
**Metrics**	**Female antennae**	**Male antennae**	**Female antennae**	**Male antennae**
Number	228676	199291	125512	126252
Total size (bases)	214371890	200247862	75777690	85751385
Shortest size	100	100	100	100
Longest size	15183	11971	12914	15057
Median size	570	608	288	322
Mean size	937.448	1004.801	603.749	679.210
N50 length	1756	1873	1251	1417
N90 length	484	512	219	255
N50/N90 ratio	3.628	3.658	5.712	5.557
