# Supplementary material for: Odorant Receptors of the New Zealand Endemic Leafroller Moth Species Planotortrix octo and P. excessana
Source: PLoS One. 2016 Mar 22;11(3):e0152147. doi: 10.1371/journal.pone.0152147 (PMC4803216; doi:10.1371/journal.pone.0152147)
Supplement: S5 Table — (PDF) [file pone.0152147.s009.pdf]

Planotortrix excessana

Gene Name	GenBank Accession No.	Length mRNA (bp)	ORF (aa)	Sex biased expression RNA seq-count	Sex biased expression qPCR
PexcOR1	KM678293	1242	MEVFDLGYIKRIRFALRYLGAWPSHVFEDC VTTKLTAIRRYGYTMLLCLMCSLGMVSQV QYLIKYKGILGFIDLGQTCLIFLISSVFFQRTF LPMQRAYRENIKDFVLGFHLTHHKNKSEFS FKMCNRVNKICEIATIVQLAQICITPFMFNIV PFYSNYKSGMLSSDRPANGTFELSIYYALPF DVNGLWYTLLCIYNFYASFNVCFMFCFHD LLICVFVFHIWGHLTIFEHELNYFPRPRKTV GSKTDPLRYSFEENKEVANGLRKIIKQYLMI KEFLANTSATYSVTLCVYYGFHLVSDCILL LECSTLDADALAKYGMVTLVVFQQMIQLS VVFELISSKGDTLVDAVYGLPWECMDNGN RRSVLILLQIVQQSLALKACNMVPVGVQTM VAVLKASYSYFMMLNTFAN	-[a]	-
PexcOR4	KM678294	1134	KFWGLLLPENTTMKYLYIFLHITIIYFTASEY VDIWFIKSNINMLLENLKITMLATVSVIKVT TFLTFQKDWVSILQYVTEADLNQRKTEDET NKSIIQTFTRYCRKITYCYWFLMYTTVIIVM VQPIIKYSFFPTYRDNVSSGKEAYLQVVSS WVPFDKSTMTGYIAACIFQSYAAIYGGGWI TSFDTNAIVIMVFFRGELELLKRDSVAMFGS ESAPVSEEEAKRRLKVCYKRHVDLVRYSRL FDSCLSPIMMFYVFVCSVMLCVTSYQITSER NLMQMVLQFEYLVFGVSQLFLYCWHSND VFYISQDLLRGPYESAWWARNVLRKDLAL LMVQFRTEIVFSAGPFAKLSVPTFISILKGAY SYYTLLNQSQVEK	-	-

PexcOR5	KM678295	1197	MTKILDDDMKFDKMFWISTRAMRINRSHP YIARDRPWRIQFTFILILSVVCFMLLLYSIRC DIQNGKFTDASKNVIMAIVAFTITYKYAILL RFQESVTSLIRIVDEDYKLAKEFSEEEQRIVL HYAMRGDKVSKFWIVSAFTTGAIFPIKAFL LMGKSYFAGEFKLVHMFELSYPWIFEDYK NTPVIFVMMFLLTLFFDVYSISMYIGYDPLV PIFMLHLCGQINILKIRISQVFSDPSDSDEIVR AKIRIIILKLQDIYSFIEVIKINFTMLYEFMMK TTTFLLPLTAFQVTESLRNGEVNLEFIGFFT GVILHFFMPCYYSDLLMETGNDFRLALYSC GWEKHSDLRTRRTILFMMARATKPLVISTV FYAICLDTFAKMCREAYSIFNLMNAAWA	-	-
PexcOR7	KM678296	1248	MDAFNLRYMKRIRFTMRTIGAWPNHEFGD HPATALSPIRRYGYTTFLFTICCIGMVAQAM YLINNRDTLSFVDSGQTLLTFFLCTVYIQRT LLPMQTSYRAVIKEFSLNFNLVHYEDKSDF SAKMSSRISKICEILCTIIHMQIYIASFFFNVV PLIINWNDGMFGPDRPANATFDQSVSYALP FNQQTYYCYVIIVVYNWYVSYNLSCVFCC QDLLIYVIVIHIWGHLKIFEHTLNNFPRPKVE SKSGINLARFTEEENKEIHYKLKEIILTYLKI KKFIETTSTAYSETILVYYGYHLVTDCVLLL ECSTLDPKALLKYGAITIVSYQQLIELSVVF ELISSRSEGIINSVYDLPWECMDNSNSKMVL ILLQIVQESVAVKAGGMVPIGLQTIVAVLK ASFSYFMMLSAFADD	Male	Male
PexcOR9	KM678297	1134	AGAGNFWYEEGEIGNDKSIKYVVLSTIMFFI YVFMTILEIIAAIFSDLPKDEKSDSVSFAMSH TIVLIKIFSVMGNRKLVKEVNYKMVKICEA YEDEKRVAENYKIMKMNVYAYVAAVYGS LACFVFEGIRKMQSGSHFITIVTYWPFFEDE SLSAVLFRFFTTCVLCVLMVTMIAIDSFVM	-	-

			VTLIMYKYKFITLRLYLENLREEFDKNNYA<br>GNEKDAAGKLQSGLIEGIVMHRDLIRLSNDI<br>DRSFGTVMALQVCLSSGSAVSLLLQIALSK<br>DLTFVAGMKIIFFVIALFFLLALFLCNAGEIT<br>YQASLLSDSIFYCGWHACPMGRDLRRLVL<br>MACASAQRPLVMKAFKMLQLTYGTFLTVV<br>RSTYSVFALFYAQNE		
PexcOR10	KM678298	1134	YVFGLWQSGSKLRNSCYNFIHFSAFLFVFS<br>QIVELWIIRHDYLEALHNLSLTALGMVCIFK<br>AITYVLWQSDWKELVECVSAEEIAQIGKQN<br>ETCAKLMRNYTIYARIVTYLYWNVTISTNIT<br>MVSAPFVKYATSPEYREQIRNGTEPLPQIFS<br>SWFPFDKSEVPGYFVAIFVHILVNIHGGGMI<br>ALYDSNAVAVMVFIRGQLAILRENCKNLFK<br>DYEQVTRREILDRIKECHRHHNFIVKQSALF<br>DSLLSPVMFLYILVCSGMICCSVIQFTSDEA<br>TAAQKIWVLQYTAALIAQLFLYCWHSNEVI<br>VECNDVDGGLYESDWWKGDVRVRKQLVI<br>LGGQLRHAVSFSAGPFTSLCVPTFIDVIKGS<br>YSFFTLLTQMQEE	-	-
PexcOR12	KM678299	1179	MPPPQSDSFRHNCFFWKVFGIWPGRTPNKY<br>YKYFSFTYLITTLVAYNALLTLNLYYTPRKI<br>DLFIHEVIFYFTEIAVTTKVAMILLKRKQIVG<br>IFELLDYEEFKGSDDTGRKIVETHNSNFKIY<br>YKILSILCNFTYLSQVIYPVIGMLIFKTTLDL<br>PICKYYFLSEETIDNFFTLIFIYQSFGMYGHM<br>MYNVNIDTLIVGFMVLAIAQMKVLSHDLE<br>NLKIEKTDTGRGEELDQKQLQKLYKCVKH<br>YALVLKYCADFQQIISATMFVQYGVGAACI<br>CVTMCALLLPLKVETFIFMVGYFFVMSLQL<br>FVPAFLGTQLLYESQELMVAAYKSDWIPRS<br>RGYRQSIKLFVERAKHPVVITGLKIFPLSLA<br>TYISIMKTAYSCFTLVRFMQDREEH	-	-

PexcOR14	KM678300	1119	MIVGMLYPTPGTDWRRAIGIAVAMASVVPI<br>SGMAALDVWNSWQRKDIVNITRHCTVIGP<br>LFGVLFKMIIFFINRAEVYRIIKTIDGDHAKY<br>NTLPERYKAVARALLSDSMYYSEYLWACV<br>VGFCVLTFPLSAACLNVYNCLCKAEPTLYL<br>VHDVEKPFSRPEERFDSPFFEIMFVYMTYVS<br>VLYIFNFTGFDAFFGLTINHVCMKIELLCMS<br>LDDAMLETDPKVKFEKMVDVIQEQNNVYK<br>MLELLQETFNIWLGIILIATMAQICNCMYQV<br>LEGYGIDPRYLVFIWGTVVHIYMPCRYAAK<br>LQAAAADIATRAYCSGWERSGDLRSRRMV<br>AFMIARAQVPVNITAFNMINFDMELFVSLL<br>QKSYSIFTLLRP	Female	Female
PexcOR16	KM678301	1143	MSLLGVWNPPKNESILRKLYRYFMLSLQHF<br>FLIFQVIYMVGVLGDLEEVSQSSFLLFTQAC<br>LCFKVTVFQVRMDSFRELLAQMNSDVFMP<br>QNKGHEEILKLQATRIKRLLLGFMISSQTTC<br>SLFALRPLFDDANRNFPFKMWMPVKPEHSP<br>QYELGFLFQLLTISMSAFMYFGVDSICLSM<br>VIFGCAQLEIIKEKLLSITSIAEKRVNKSTDV<br>NSILEDNYRVLIECVAQHQAVIKFVKLLENT<br>YHAHLFFQLSGSIGLICMTALRILVVDWKS<br>VQFVSMVLYLSVMCSQLFVCCWSGHELTV<br>ISSELHTTLYQCCWYEQRTRFKRALLFAMM<br>RMSRPLEFRAGRYVPLSRQTFVTILRMSYS<br>YFAVLNQANSRNEILEH	-	-
PexcOR18	KM678302	1185	MSEKNVALFLNRPRNILGFFGIWLPPASFTL<br>LYTIYMLFILLTQYSFVLFEFIYIVEVWGDL<br>DAVSEASFLLFTQASVCFKVTRFLIHKDKL<br>VMLLEFMEEEVFQPQNKIHERYLITLSTRV<br>KRLCLFFLMSACTTCTLWGMVPVVDSTAG<br>ERFFPFLIWMPVGPANSPQFELGYFYQMAA	-	-

			IYISAFLFIAVDSVVLSMIMFGCAELEIIMNK VEQIQKVPLSGKLKQDERDQRIRDNCELFK KCIKHHQEVIRFIDIVEDTFHANIFFQLSGSV AIICIIGLRISIVEPGSMQFISMLNYMMTMLS QLFLYCWCGNELTIRSELLRDVMYLSPWYE QSNPFRRLLWVAMERMKKPILFKAGHYIPL SRPTFVSILRSSYSYFAVLNQAQNKEK		
PexcOR19	KM678303	1152	KDFNETFKFCSFALAIGLIYPNKRNVRKRVI LFLYVLLFNTFTLFWMFWYTFKCLVNHDV YNSTRNITLGVIVFLFIFKTCYVNMKTAMFS QVLKQITKDLLKGNEMEEDYQEIYDHFIKQ GKLGQSVYVLIPCMLSFMFPIYSGGAMIGG SLKNDNFTKYMIHEMDLKYVEDKQYHSPY FEIIFAYFTTPCFVLLPNYVGFDGSFCIATSH LCLKLKLMAHSIKKAFEDSKSTTELKARLKI CIKDHQEALEFHVLIQKTYGGWLFAVFVLT SFLISCNLYQIYLVGIDPKYTMFAITGVFHM YAPCHFASNLIAVGDEVCSDVYCVKWEAW GDPSITRLLVFVMARAQKNVQLTGLNIVTY NMDTFVSLMQTSYSFFTLITR	-	-
PexcOR22	KM678304	1158	MAIAMTFLRLANSTWWGLPPTLKLLKIHG DMFLIVAPICLTLQVVYLYVYFSELSFTTLG TMFSMIPATLVVNVKLIVSMRSAYKQIMYN LMDKIHIYNFLDDNDDFIKTKLLKVERNTR WLTTFLVGFILMDWLLWSVIPLRNNIINKER IGNRTIRLETCLYMWMPFDYGYEFRTWLIT HAMNVYLVFTGGLVLSFYDSINFIFIFHFLC HVDILRHKMRTYFAVKLNDTDTKGKIVEII KYHSFILSTFKEMEAGFGANVTLNYLQNLV NDSLLLYQIMVGDKANRFTYLCMMQFHM GGLILISFALEQIRIKTEDLALDLYQAPWEK MSTSNQKLLIPILLRMQTPLVFEGALGLQTG VRPLASIIKSTFSYYVMLKSSIE	Female	-

PexcOR25	KM678305	1134	MFGNLFELSSGIFAFNLKYLFIAGLWPSDK WAKENLFIYKIYEVSLHILGATFLIVTGIGTY RSKNDMMILLGNIDKSLAGYNLLAKVIIFL YKRKELENLISEISRSGDQISEERKKLMLIHV VVLTGMIMSIAGLFSGLAFYNNEMTIEAW MPFDPMDSKMNLMLSMQILIITFTVPCVWR GIAMQGVVVSLTMYVCDQLVDVQERLRAL VYTPDSEFAMRAQFKDIIKKHIRLVGYSRT MTDIFNEYFLIQNLAVTVELCLSAFMATIAG LEQQSLLASFLAFLCVALTNAYIYCYLGDE LIVQSQGIALAAYESTWTSWPLDLQKDLLIL IRAAQKPLILSAGGIAAMSMQTYSQTLYNG YSIFAVLNDAEIK	Female	-
PexcOR26	KM678306	1170	EGVPLSYLSLVPHLKNLRYCGILPLPSDNPIL KRRLHKIYMGFSFSFIMLYTVQQIVNVYQV RSDVDKVMDSMFLLLTFCDSICKQVAFIMN PHKIQEMLEIMQGPNFNQGREEHRPLLLKT VFQAQLLLKIFNTLCLLTCLLWVLFPIISHFK EEAIEFTIWLPFDANQNPQFYVAMVYVWL QTSWLGYNNSTMDIFIMYLFAQTKTQLTIL RIDLENLVSRCKTEAAVSSFTYTDLLEKHFR GIIHHYNEIIKFSSMNEGIFNGPILFQFLISGW IICTTAYRTVNMSPASGEFSSMILYMICILSE LFLFCFYGNEVAYESEILMESAYCMDWTE MPVKYRRFLIIFMERIKNHIKPKAGKIVPLSI NTFVQVVKSSYTFFTFLNKA	Female	-
PexcOR27	KM678307	1209	MLKKYVSRLQDPNHPLLGPTLWGLQSWG MWQPNSGLSRIIYNLIHLAAILFVLSQYVEL WLIRSDRESALRNLSVTMLSSVCVVKAGTF VSWQRHWRDVINFVSTLERRQLKKTDKTT HDVIEGYTKYSRSVTYFYWSLVTATVFTVI	-	-

			LAPLGAYLSSAEQRELMGNGTIPYPEIMSS WFPFDKTKGVGYWVAIVEHSLICFYGGGIV ANYDANAVALMSFFCGQLELLSANCKAIF GDGSGLVSYSEAMNRIKECHLHHLSLIKYS KILNSLLSPVLFIYVIICSLMICASASQLTNQS TTTMQRVWVAEYLVALIAQLFLYCWHGN KVFFMSERVDRGVYESEWWQCGVRLRRC VLLLGGQLRRTILFAAGPFSTLTVATFVAIL KGSYSYYTLLSNNED		
PexcOR29	KM678308	1131	WLLNKIGLILPDNTWNRIYYVLIHEIVAFFV FTQYMELYVIRSNQELVLSNLKISMLSIVCV AKSNTFVIWQNNWRQVIDYITKADIFERDN QDKAKGNIINSYTKYCRRVTYFYWVLVFTT FITVITTPLLIWYSSYDFREGYKNGTEAFPHI FSSWMPIDKESAPGSWITVIWHTALCAYGA TIMAAYDTSVMVILVYFGGKLDLLRIRCKE MLGVIEEAISDKDAEAAVSQLHGIHVLLLK HSRMFNSVLSPVMFVYVVMCSLMICASAF QLTSATNSTQKILTAEYLIFGIAQLFMFCWH SNDVIHKSQQVTMGPYESQWWAVGLRQR KSVLILQGQLQIMHIYSAGPFTDLTLATFVTI LKGAYSYYTLLRD	-	-
PexcOR30	KM678309	1188	MAILKFDDLYALPVTCLRLIRAHPSIPRDRS WVWQVALMHGLFSVGFSSVVYSLIAVDIK ANDFIGTCSNGIFVVVWVVITFCYGVILIFS DELNTLMRLNQGYFDAADSLPEEDQAVIRE YAFRARWVARLWGFACFLNGLLFPLKSVV LTLYSIFIDEFRLVPMYEVSFPPAIENIKETPV MFCVIWVMFAIYAAYASIMYMGFCSLGPIF IINTCAQLELMAKEFKNMFNITPYDDKLVG VKLSAVIKKSQDIKANVQIINDVFRLYYEVT LKAAAIMIPITLYLVIDTIKQGELRIEFLMFDI TATMLCYIPCYYSDLLMEKGEAVRLAMYT	Male	Male

			SGWENNFDKKARASLLIMLTVAARPISISTI FRVVCLDAFAGLCHEAYAIFSLINAMWN		
PexcOR32	KM678310	1251	MKKPIGLQLNRYLPVFNSGPPKLVNTIQRL VWNTGLWSNQSLGLHWLAKLAIVCFFSTN FTQFAAMIIERDDSERVFECFSVLSFCGMGA LKLVNLYFNRERWLFMLSQVTLLENKQLD DASDKQSVEYDSDDEEECLSPHIERYTRKH TVTSNLLLMLYSATAVIFIVTPFVEYALQKN PKAYPHILPGWAPLDAVGFLGYFVSVLFEV VGSIYCVFVHVAFDCTSVGIMIFICGQFSML RYSTENIGGRGQDCRLSAARDARARSRIAK THGTHIILCNVINELGASLRNILGVYFLVAT LTVCSVAVRINSQTLSFMQLVSLLQYMCGT LTQLFLFCRYGDAVFHESYFSMGQGPFGAA WWGLRPRVRRELAMLGAAMARPRALRAG PFNTLDLPSFVQASYTFIYLYLLATG	Female	-
PexcOR35	KM678311	1233	MEMSNCHRDEMTRTRKKEMSRFDIRNIFKF LEDPEHPSVGPHLHLLRLTGQWHPNDSIRIR LKLVVFFVIVLLFSSQYVKCFMGIEINSLLLI LQYAPFHMGIIKTIFFQKDYSKWKELIGYM SSTERRQLNEDKSEVEQVMDSYIKRGRRVT YFFWALAFFSNFTIFSEPYQKNQTVENGTD VYVKIFNFFTPFNQDVPPGYYVSMAIQTVL GHIVSAYVVAWDTIVVSTMIFFAGQLKISRI YCVRVIETGSAEQSHRNIVMCHRFHGSLVK YQKLFNSLISSVMFVYLVVVSVNLDVCIIQL SQLKDDLAALVASLVFVVACLIQLLIFYWY GSQVTEESVLVSYGIFESDWFKTDAKLLKE VLLLQLATSKRLVFRAGPFNEMSLTTFVAIL RSSYSFYTLLNETK	-	-
PexcOR37	KM678312	1149	RESMAVTKARLKENSLDSLVWLVNIVPRIL GFDSAREKVFIPYWILHFSLFTYVYVVGCV	-	-

			VYQARHAKVASDFVKNYVNVSLVILTVMN SYWFLVERGLLKTVLKKVKEIDNYVVQSG LFAEKHTHSLFLIKRIIHIFGGLNLISFFVVNI PNRLDLKIETFSMAICVGIEPLTSSPNREICK VLLGMTECSLGAVTLHFLTLMLILIAHTTS MYQILSDDIMMFNTELEDHRDYAYIKDKLS MMVYRHALILEITQGLKSLYSMPMGVNFG LNAVCMCFFCFLTSDEYLGFMPILVYSFLAF FLYCLLGQRLTNAAEVFARAVYSCGWEKM NVKEQKAIYIMLMQAQKPVELLAADIIPVN MYTFASTIEAIYKFTTAVKL		
PexcOR38	KM678313	1266	AVLLRALALDVDGRNTTGISPLNYLFMLVT FVFYFYTYHLSTIWFVFWRADGDLLFYILL LSLSISSLIGVIKLLYMYVSSKKLRQIVADYL LCDAAIIPGSRLANNIKVTLRKVKRRAAIY WLIIIGNGVVYVVVPLLTPGRHITVDEQILW GLDPMFESPNYEIATTILWFAVFLTVYAPA NITGFLIIIVGYSEAQMMALSEELLHLWDDA QNHCQRLPEDDNLPMEASSSSGTNFFARRS KDSIILSGPAFKENETTAPETSVQEKTINGFI KQRLVSIMKMHAVNINLIKRVENIFQNAMA LEFTLLSGGLIAELLGGLENTYVEVPFALVQ VAMDCITGQKLIDANAVFADAVYGCKWE NFDLSNMKLALVMLQNAQKTMKLSAGGV NSLNYSTLMSVIKSIYSAYTALRSTMNT	-	-
PexcOR39	KM678314	1188	MISNVAMMLRVVALRIDPRNKKPIPTFLYIL TSVCVVCYIYNYQISMVWFVFSRCQETGDV VAAMMVLSTGTTSLIGITKLFTVYYYQENV QSMVGRYIEYDTTMIAPASSALMLATMRN VKKRALLFGVVMMGNGFAYNLQPLFKSGR HLMDDDQILYGLEPMLETPNYQIALVNIIFS VNFICFIVSNITGLLIITTGYSESRLLALSLEIQ DVWQNALQHYRDNFDANYTEDKYDETAT	Female	-

			VELNKYVKHRLFHIQKSHFTNINLVKSIEDI FRQAIVVEFFLLTLSLIADLLGGLENTFIIMP FALMQVGMDCFTGQRLMDASFVFEAAVY DCKWENFDASNRRTVQLMLQISQKTLILSA GGVTALNFACLMSMIRSIYSAYTALRSSMN YK		
PexcOR42	KM678315	1143	ETPTFAEVFKQIKTNFSLMGIPRDKSMLNVR FYILIAFLIVMLIEEIFFFVKKMAPENFLELT GLAPCICVGCLSVLKIIPVACHRTTVFSLAS KLSELSTEILKDPKNEKVIKGNINSVRTLIKY YFILNLVLISVYNFSTPFYILYHYIASREEIFF LPYPVLVPFSTETWVNWFIVYLHSICSGFIC VLYFTTVDGLYFVLTSYVCSMFEVLSKDIK EIDNDTTDLKEIVKRHQYVLILAEDLEVIFT LPNFFNVLVGSIEICALGLNLMIGEWGDVP GCILFLSSVLVQIFMISVFGEKIITESTKVSDS AFLCKWYEMNAKSKKTILMLMIRAHKPQR LTANKFSVICFNGFSKIISNSWSYFTILWTVY SRKNEQ	-	-
PexcOR44	KM678316	1272	EYTKEKLSFLTPFLPYGVLESWDGLDPRLY HAVHIYWLKFYGLWYNTVSKCSFQFWTHI VYGFLVLWLVCFLPGIGEVVYLLRRRDNIG EIAEGLYLFLSEMYTYIKLSALWLNKKKIID LLEYLHREEFKASEVEHREILRKSIKTARFV MTYYSTICVGAVSVAIIMPLAEDFEVLPTNV EYPYFDVYKSPAYGIIYIHHIYYKPATCIIDG VMDTILSAFIASAIGQIEILAFNLRNFNLLAE RRQKRSLMGDINNEESTRYHVRAVFKDIIK HHNSIIRYVSLIESAFSLASALQLMLSVMVL CLVGIQFLSIEEPMSHPIQIAWMAIYLTCMLI EVFIICWFGDELIWKSNELRQAAFDAPWPT TDPKTAMFIIIFMERCKRPLRVTAGKIFTLSL DTYTNLINWAYKAFAVMRKMKK	-	-

PexcOR45	KM678317	1248	MDAFDSSYLKRIRFSLRTVGAWPNHVFEDL<br>PTSKLAAISRYGYTSFLFTICCIGMVAQAMY<br>LVNNKGILPFIDLGQSCVTLLLCLVYIQRTT<br>LPIQTAYQETIKEFVLKFNLIHHKNTTDFSA<br>KMYDKVNRICEITSIVAHIQFLMSAFMFNIA<br>PLCLNYRAGMLSSERPANVTFDYSVYYALP<br>FDQNNGIWFTIITVYNAYVSYNLACIFACH<br>DLLICLFVFHIWGHLTIVEHKLNCFPRPRIH<br>VGSKIVPLRYSNEENKEVAAELKEIIKYYIM<br>IKQFITKTSDTYSVTLCVYLGFHLVSDCILLL<br>ECSTLEVEALAKYGVLTLVVYQLLIQLSVV<br>FELISSKSDCLPDAVYGVPWECMDDGNRRS<br>VLILLQIVQQPMPLKACGMVPIGVQTMLAI<br>LKTSFSYFLMLRTFANN	-	-
PexcOR46	KM678318	852	TIFITVNYTSFLSCDRETRLQQLLYFCLTTVT<br>VAGWAGSAEKNQLPLRAWYPYDTSKSPAY<br>ELTYVHQVGALFIAAYLNVGKDTLVTGLIA<br>QCRCRLRLLGLGLRTLCEDLVPNEQGILTPE<br>QEKTAWSRLRVIVQRHQAALEASSLLQDCF<br>SAPIFAQFMVSMVIICVTAFQLAAQTGNLV<br>RLFSMGTYLLNMTFQVFLYCYQGNQLSEES<br>IEIAGAAYECPWYACSTQLRRGLLVVMVRT<br>RRAARLTAGGFTTLSLTSFMAIVKTSYSLFT<br>VLQQAEESKK	-	-
PexcOR47	KM678319	1362	MPDSKAADRPTRYFATHFYLLRFLGLGWW<br>HHPDEDNTSNFPGLYIYYTIVTEIVWVAGF<br>VGLETIDPFIGEKDIDRFMFSLSFVITHDLTII<br>KLYIFLFENRAIQDIVRTLEIDIYNFYQNNER<br>NRATIRITKIMTGSFVFFGWITIGNTNVYGTI<br>QDIRWKAEVALLNDTSLRPLRTLPQPIYIPW<br>KYQTDVSYISTFVLETVGLLWTGHIVMAID	-	-

			TFIGSVILHMSSQFVILQEAFMTSYDRALKQ LLTEIPHSMDARENIIMPNEDDDNTRKDSLD KIEAKVKSFYTDEQIESAVEKSVLNCIRQHQ VLISCVEKFRVTYSYGFMTQLLSSMAAICV VMVQVSQDASSFKSIRLVTSLAFFVAMIIQL AIQCFTGNELTLQTERVSDAVMQSKWERM TPRVRRYLLLAMLRAQRPLRLTAAGFAYM DNGCFLAIIKAAYSYYAVLSQKEV		
PexcOR48	KM678320	1194	MFREFLDRLRTEEMPLLGPNTWFLKTIGLR LPKGKIKKVFCVILHEIVTFFVITQYIELFSV TDDVDTMITNLKSSSLSIICVIKSNTMLLWQ DKWLEAFEYVTEADKFERVTGNPARDKIV TRYTKQCSLVTYYYYLLLFFTNFGVISLNLL VNMTNVEFRAALNNGTVPFPHIFSAWTPYD RNTFPFTWITVVWHVFITTTGAVIISAYDTA AIVLLTFFGAKFELLRLRCAELFQEGEPVTE EEFDERVRQVHTLHTQLVKNIRLVDSLLSPI MCLYTVACSLVLCTSFYQLTFSTSISQKLML AEYLAFGITQLFLYCWISNDVLEKSSKLML GPYESRWWAGSPRQRRSVLMLAEQMEKA HVFSAGPFTNLTLPTFIMIVKGAYSYFTLLR N	-	-
PexcOR51	KM678321	1173	MADILKEIHPQKYYLRLVSRLMFCFGFGTY WYEGEATRYPRLYTVWCIIIQSYIFLVVLDL ILAILRPDLSDEEKSAVIQVGFSQLLVILKFII VVLQRHRIRDGFKKLLEEDRDIFTSLELEKE SVKTAKVFFLSFMIASYSYLTLNLVWSTFV SIMHGTPIQTQITYFPTPALTGFPYNLLRILII AHWWTHATMMITADCLCSLPIFFVTAKFK QVQMYFEILGENNLEDWSGEEFRKGFVNGI KLHQNALWCASNIQSALGILYGVQIWQTVI LIGITLFQLASVERTMSNMITGLVFIVCVSLL TGAYMINSGIITYEAAKVATAMFHCGWER	-	-

			TPADRRLRSLLVAAVQRAQRPVYMTAFGM MNLSHESFVTVLRAAYSFFALVY		
PexcOR53	KM678322	1140	MQPSDCFKISFFVLIVAGVWYSPSWEKSRY LHAVNIYRVFAMIMILIGMLSIQFVYFFTVV GVDLDKTIDATALFTFVGHLYKAITVIINRR RINKLLEIIDSEEDKDDLMQSMAEKISFVSKI YNGGAGLTAVMWNLIPFTKPTLTLPFYYPD LPSDSPWFVRWYAYQTVILIINGVAQTSAD HVFGGLMAFAATQLKLLQYKLETIGSRKET EELDAAQREQDDYKEAVSCVRFHLKIIGVV DELTSIFGAAAFGQFLLAAPLLCLSAFILTTS SDPTEIITRLLYFGCISGQLFFYCFCGNMIKI QSDLVATAAYNCSWETTSVRTQKTLKLLIL RGQKTLSVVAGNLFELSLVTFGALLKSSYS FFAVLNKKHND	-	-
PexcOR54	KM678323	1128	MKNSQCLSSSIAVMKYSGVWKPDGLTYRW EMAYKIFGFVTQVLFFYFIILAEIGYVYTFL DNVERMVDAAVLLLSHLVQAVKVMTIILR QGGIKRMIEMVDGPAFSKSDPKLKAILESST RLAAMVGNVILCSACTTGIFWGIVPALNPE LTLPLRTAYPFDIHGPYIFPAMYAYSSISVIV AGVGDAAENFLVSGLFTLASAQVDVLREE LKAIGADGCTDNFEKATLCVKYHQRIIGYV EEMAHIFGLPMFCQFVTTSVVICMTIYKITV TQEPVEMVTMVFYLMCVFMELLMYCYPG DVLLNKSLLVSEAAYPNDWSNDKRTSRVL LLTALRAQRPLAIDAGGMFRVCLPTAAAV VQTSYSYYALVRQQLAKE	-	-
PexcOR57	KM678324	1212	MYPSFQAFREDFDALALAGYFKIVSKPVSR LKRSLHDAYRTAVWVVVITYNLQHVIRVIQ ARHSTEQMVNTLFVLLTTLNTLGKQIAFNA RSARIDRLVEIIEGPLFASRNAYHEEIMKAH	Female	-

			ALEMSRLLKLYHAAIYMCGVMFGVFPLVNRILGEEVEFTGYFPFHTNDVLPFALALSFNTIVITFQAYGNVTLDCTIVAFFAHSKIQLQMLRYNLEHLVDRNWTKLDTKINNTSVPKTFIDIEDAAFGELLRKRVAHCVEHYKIIVWFTNEVETVFGESMVVQFFVMAWVICMTVYKIAGLSLLSAELVTMALYLCCMLAQLFIYCFYGTQVKYESEFINHSLYCGDWLALSPRLRRLLLVMMVRCSRAVAPRTAYIIPMSLETYIAVLRSSYTLFTFLERK		
PexcOR58	KM678325	1155	ICKVIFLSCATNFGFEDTNLPSCFIRIYGLISKILEVIVFVFLASEWGSFYTQKNLTEKQLSDLYLFAFSHVVLYAIYASVLYHRERIRELVLALTVTLKQVCNDEATERVMIMKIYRSLTAMVFVCSGSLFSYGLDAGVQAFTSNATFTTVIPAWPDVEDRQFIASVARIIYYIIWVIFVVRVLSIYLIVLCITICLGHQFTNLHKYFLKLNDIFEENGSQEDKERRYEKAVKVGVKMHSITLWCAEQTQVTCGVAYSGQVIINVSVLVLLMIQMVHTERTLTSAAPIAMLGASVLVSSAVFMLNAGDITIEASRLPTAMFLSGWHNCRGQASVRARKLLTIAMAEAQNPVVIIGLGVIQLSYQSYVSIVKSSYSLFSVIYSN	-	-
PexcOR59	KM678326	1185	ELRGEINETLSLCLFCMRWIGLSFEPPTSTRAYLRQKLMFAVSVCAIVYHVFSEIVYIGLTLSNSPRVEDVVPLFHTFGYGALSIAKVFALWYKKDVFAEHLQELSGIWPMPPLDENAQRIKETSIAALRLVHRWYFAMNMGGVLFYNVTPICVYLYELWSGQDAVVGFVWVSWYPFDKYQPINHVFVYIFEVFAGQTCVWIMLCTDLLFSGLASHIALLLRLLHSRLETLAETKKTNEEYYQEIIGNIKLHQRLIRYCNDLEEAFTIVNLVNVVLSSVNICCVVFVIVLLEPFVAVSNKLF	-	-

			LGAALIQIGMLCWYADDIFHSNADVALAVYKSGWYRTDPRCRRALIFLIRRAQKPIAFTAMKFTNLSLVTYSSILTRSYSYFALLYTMYNNS		
PexcOR60	KM678327	1218	MSETHDTSRKEIHKSLDLLNFCTRMIGLSFIDDPPTSVFAKIKSSFIFGLSALSLMLLFTVEVSYIVSLVIKTASLADLVTGLHIVGYGVMSTSKLLTLWVKRHTFRKSIKDLAEIWPEAPENQEEEDLKKGSLKALRIGQFSYTFFNVLGVVIYNITPIGIHLYRTSRGLPSVMGYVWFAAYPFDKTKPINHAFVYAFECFSGACSMWSMVSTDMMFTTMASHISLLLRVLQIKIRRLGFADAPTPTDNARSYQEIVNLIKIHQRLISFSDNVEDAFSLVNFINVMISSVNICCVMFVIVLLEPWVEMSNKFFLVAALIQVGILCWYADEIYQASVGVADAVYASDWYNGDVRARRALAVFIQRSQKPLYFTALKFRPITMITFSSILTTSYSYFTLLYTVYSD	-	-
PexcOR61	KM678328	1221	SCHDEENFQNDIDYVATVASRICLYPFYGRPKYKIFCYYSICFLIFFVSAQQFTALCVYGFKSFLDIVGIAPNIGVTLMAVTKYIKVHRNKHTYNLIFNHLRSNMWDVVDKSSQENRKILITYQKVLKFIILWYVYYIVPLLSIIVTFPLLIMYYDCKVLGKDLELRYPFEAWYPFDKIKWYYAAYTWECVMTAIVVYIYTYSDTINVSYIGYICMELKLLGTHLRHLIGTKEIIDLKNSHEVAAVHDKIKCKLRRIILKHVFLANIVSQLDGILGDIMLVNYTLGSIVICLTAYTFTVVDEFYSTVRFFFFFISFIISILNQCVMGQVISDHSEGLAEELYNSEWTYGDRDTKQLVLILIMRMQKPFQLTAKKYIAMNLHTFTAICSTSYQCFNLLRTMYDPKKN	-	-

PexcOR62	KM678329	1068	MTLFGAGETWYLVSNYHLLDIFIEQLNVMVIQWTAIIRFKSMRRHKDIYKKLAAAMESSNFDTSTPARLALVEYWRLRSEKYLKVIYGLGTCTLAAWFVYPLIDDVECNLMVAVRLPLDFCSPVRYPVAFLMTMVAFCYVAYYVMTNDVIMQTHLMHLLCQYAVLIDCFENILEDSERNFKGVSRNDLIHNNDFRITYLRRLGRLVDQHKLLLKHTMDLRQSLSAPMLAQVMASGMQMCFAGFQVLMTITDSITKFLMSFLFLGYNMFQLFVLCRWCDEIKTQSTKIGDALYCSGWERGLTTIPGVRRRLLLVAMRANKPLVLTAGGLYDLSLSSFADLVKTSYTALTVLLRLRHD	-	-
PexcOR63	KM678330	1185	METAFFESAYRVIYITGWSSCDRGIGYQLYCNSIKLLMALFVVGETWYLASNYRSLDVFVEQLNVMVIQFTAVLRFKSMRDHEHIYKRLAATMESPTFDTRTRARQALVEYWRLRSERYLKLVLGLGTCTLAAWYVYPLIDDVECNLPVAVTVPVDYCTPVLYPVVYLTTTIAFNYAAYFIMTNDVIMQAHLIHLLCQYTVLVDCFENILIDCEENFTGVNRSSLIHNNNFRLKYLKRLGRLVDQHKLILKHTMELRKVLSSPMLAQVTASGLQICFAGYQVAMTITDSFTKFLMCFLFLGYNMFQLFVFCRWCDEIKSQSAKIGDALYCSGWERGLTTIPGVRRRLLLVAMRANKPLVLTAGGLYDLSLSSFANLVKTSYSALTVLLRLRHDS	-	-
PexcOR64	KM678331	1290	MTIVNNVKSFVNKEGFDWDRPDMTLQIFHPQLEMFFAANGIFFNNRESKIRFIWPVLCALLSLVANSFEIMFIWHGVAIKDYGFATESFCYFFILGSIVFVYFSMLTNRKKIFLLADNLNKDFLFICNLGAHYRHTFLKGQLLIWKLCWAWLSFALFIMILYVGNTLIALLYQSTLATQDEH	Female	-

			MVRPFMFPIWLPADDPHRSPNYEIFMSLEII LIFVVFCSFIFYVYILFHLLLHYYNLMDMILI AFGELFDDLDESVVTLPTEDPRRKAVQAEL NRRMGQIARWHHSVFESVETISSVYGPALV YQTMFSSVVICLIAFQVAEQLSEGKFDYLFG ILGVGACMQLWIPCYIGTLLRNKGFSVGDR CFYCGWHSTPLGRLLRRDFVIFIQRSQEPLA IKFTALPHLQLETFSSIMSSAYSYFNMLRQY N		
PexcOR66	KM678332	1203	MTKYPKYKKSKATDFFFNLNRVIFFCNSLN FWVEEVGVSALFVKFYQSLSKLINIAAYIFV AFEWGAFYTQNNLTEKQKGDRFLMSFSHTI LYSFTVIMIHHRERVRELWFTLAVTLKKDF NDAETERLMIKTTKFYTSAFVVICGNALIFY GIDGIIQLAYSDGTFVTLIPFWPDVNDHRTV ASAARIATYVFWWFFMARITSVYLLVLTITI CLSHQYTNLQLYFKSLENIFKQNLPQSIKEA RYERALKIGVKLHATTIWCTQQVQETCSM VFSGQIIVNTTVMVLLLSQMVASERTLGNT LPIAATIVSMLFSTGLIMWNAGDVTVEAAR LPTAMFLSGWQHCQDKASYRIRRLLLIAIT QSQKPVVIRTLGVIELSYQSYLSIVKTSYSIF SVLY	Female	-
PexcOR67	KM678333	1278	PLEFSKPFAICFDLLAKANISVHDQKCSVRR KMRSLLLVICYVTFYVSLTVSFSKVFTGVL GFYDLANLLPIFIVATQGAMKGAVIITNLSK ARTLIDELGAMWRTSGLTRNQLARKGMML KRLNLCNAVFYWMNIVGTWQYILVPLFET LFRTFVLGQDKQLFPFICTFPFDPMRNWLV YLLTYFYESYSMLHLIYMYLGVEFLMITLC SHLATEFELLREELLHARSRKETIDVLDYVN CSGDIENGDSIVEHDTIDIDDGIMLDEDRPDI KDVIRRHQKLIMLSELLDDIFNKMIFFNLLF	-	-

			ATITICFFGFVAKIARDLPEMANNFVGVIAS MIPIFNLCYYAEMLSGASAGVADSAYHNL WYEGDLRYQRIIIFIIVRSQKACSLTSMRYSP VTLNTFTTVLSTTWSYFSLAISVYETDKQ		
PexcOR68	KM678334	1164	KIFKIIGLDFLGDPSVANHCRHVSFVSMLLL FFTGQLLFFFKSDEIGADFMDIANAIPLFMM AVQDLVKIVALSKMQRIKGIIMEVAELWPN EINNEEKKSIMNSWIWNLKMFNDCVYKFV AFAVFVFVWGTFFVTVFTTSDGVITYLYSF QLYYPFKIDSMWKYSAAFLFQSVTGTIIHLC LYQPCDLLLFTLTVDICILMRLLQYDLENIR VVGKDRNGVFDPAEAEKSYRAVIELARTH QKLVKISENLNEVFGTIIFTVVSLSAVILCFF GFLLITVGGTQYQMLRSFLAVFVKMFIVFC LALPGQILSDASCGVADAAYKSLWYESDL KFRKIIFIMIARSQKPCFLSALGYSQMNFNTF CKICSSSWSYLSLLNQMYQDTER	-	-
PexcOR71	KM678335	1332	MTVDSKPIEISDYTHFIIIPLQIVGCWDWFPN PDKQYKILINNIYLALVLFVLINFPTTLIVNL YTEWDNVMGSLEMLADGLPLLVAVAVVIY FALYKKELYELVEFMNGNFKFHSARGLTN MTMEHSYKSAKNFGFVYTACTLFSVTMYV MLPMIVHLWTKQPLQNWMYTDIVQTPFIV MAFLYQCLAQAFVGLAVGQLGVFFAANAI LLCGQLDLLCCSLRNARYTGLLQTGVQHK VLLQEYAGIVDDERHNYIYSETEAKDSEYH YDAKVTSYFVDRRSEFDIYSAEFDAATAEA LRECARVCQVVATYKDKFEDFVSPLLVLR VVQVTLYLCTLLYAASVKFELTTVEYLAA VALDIFVYCYFGNQIILQASRVSTAAYQCA WPAMGVRPRRLLLNILLANKRPVAVRAGR FLPMDLHTFVVIIKTSFSYYTLLDKINN	-	-

PexcOR72	KM678336	1104	EEMPWLGRLSWLQFLVYVLAVVSHTGGVL ERMGDGADMVQLSGDLSATLVLWQVTVL YVQIYGNRKLIRNLILNLGSKWSSDDHLSPE MVAVKQQSVKTIYKWITYFYKVLTVFMHL YFCLPLSAAAVKHFVLKEEFAFATIYKLKM PFRYEDNFFLYWIVYMIDYGVLYNTGFLITS DLLLVNVSMNHLRTLFIILQDDLKSIVHSAS EPFERTATRRLKEIIPKHANLLQLMVELSEA FGAIFLIHLAFFSGTMCFFGFAARVHCSPESI KNLLASSFILICIYSCCSCGQYLTDSSLDVAN AAYEGSWHLMSHEYRICILFIMLRSQKAYY IKSTSFSDISLQSFTKILNVTWSFLSLITKVYE E	-	-
PexcOrco	KM678337	1422	MMGKVKTQGLVSDLMPNIKLMQAVGHFL FNYTDENGGMSMLLRKIYASTHAVLIVVNF LCMAVNMAQYSDEVNELTANTITVLFFAH TVIKLLFFALNSKNFYRTLAVWNQSNSHPL FTESDARYHQLALNKMRRLLYFIGSVTIMA VVSWITITFFGESVRLIADKESNDTLTEPAPR LPLKTWYPFNAMSGTMYIVAFVYQIYWLL FSMAIANLMDVMFCSWLIFACEQLQHLKAI MKPLMELSASLDTYRPNTSELFRASSTEKSE KVPEPVDMDIRGIYSTQQDFGMLLRGAGG RLQNFNNPNPNNPNGLTQKQEMLARSAIK YWVERHKHVVRLVASIGDTYGTALLFHML VSTITLTLLAYQATKIDGLNVYAFSTVGXX XXXXXXXFHFCIFGNRLIEESSSVMEAAYS CQWYDGSEEAKTFVQIVCQQCQKAMSISG AKFFTVSLDLFASVLGAVVTYFMVLVQLK	-	-

Planotortrix octo

Gene Name	GenBank Accession No.	Length mRNA (bp)	ORF (aa)	Sex-biased expression RNA seq-count	Sex-biased expression qPCR
PoctOR1	KM892365	1242	MEVFDLGYIKRIRFALRYLGAWPSHVFEDCVTTKLTAIRRYGYTMLLCLMCSLGMVSQVQYLIKYKSILGFIDLGQTCLIFLIGSVFFQRTFLPMQRTYRENIKDFVLGFHLTHHKNKSEFSFKMCNRVNKICEIATIVQLAQICITPFMFNIVPFYSNYKSGMLSSDRPANGTFELSIYYALPFDVNGLWYTLLCIYNFYASFNVCFMFCFHDLLICVFVFHIWGHLTIFEHELNYFPRPRKTVGSKTDPLRYSFEENKEVANGLREIIKQYLMIKEFLANTSATYSVTLCVYYGFHLVSDRILLLECSTLDADALAKYGMVTLVVFQQMIQLSVVFELISSKGDILVDAVYGLPWECMDNGNRRSVLVLLQIVQQSLALKACNMVPVGVQTMVAVLKASYSYFMMLNTFAN	-	-
PoctOR3	KM892366	1233	MEETIRTFHGVLSVAGVTIYSKNTPDSKLWLTLQIFNVIVGFFTIVFTTCFVIINVSDILVCIQGACIWTTGLIMFISFGVCLVFRKQFRLFLIEMGFKDAMLEMPLIEYVLKLEQGERLNELKGMVIESQEKLLKLTRVLLKTYVGSVWVCASLYLSDSVYQMVVRKDDSLRLMGFDMWIPWSLQNFNVYVATFAFNAYSGYLCCIAYPGLQLTIIMLVGQTIRQLRILTFILLNLDELVLEIVGQRGENWQQHCTEILTQCVNHYIKIKRFSNKLNVICQPFYLALILDAILLVCVCSVKIAISDKSSPDTIKYYVHELCFILVVLMFCLLGQQVENESQKLEAAVTEKWYIFDRRHKVNVRIFKMALSQRMPIYIFGSITLSAPTFTWFLRTGMSFFTLVMSVFDEN	-	-
PoctOR4	KM892367	1209	MLKLISNKLEDPNRPLLGPNVKALKFWGLLLPENTTMKYLYIFLHITIIYFTATEYVDIWFIKSNIDMLLENLKITMLATVSVIKVTTFLTFQKDWVSILQYVTEADLNQXKTEDETNKSIIRNFTKYCRKITYCYWFLMYTTVIIVMVQPIIKYFFFPTYRDNVSSGKEAYLQVVSSWVPFDKSTMTGYIAACIFQSYAAIYGGGWITSFDTNAIVIMVFFRGELELLKRDSV	-	-

			AMFGSESAPVSEEEAKRRLKVCYKRHVDLVRFSRLFDS CLSPIMMFYVFVCSVMLCVTSYQITSERNLMQMVLQFE YLVFGVSQLFLYCWHSNDVFYISQDLLRGPYESAWWA RNVLRKDLALLMVQFRTEIVFSAGPFAKLSVPTFISILKG AYSYYTLLNQSQVEK		
PoctOR5	KM892368	1200	MTKILDDDMKFDKMFWISTRAMRINRSHPYIARDRPWR IQFTFILILSVVCFMLLLYSIRCDIQNGKFTDASKNVIMAI VAFTITYKYAILLRFQESVTSLIRIVDEDYKLAKEFSEEE QRIVLHYAMRGDKVSKFWIVSAFTTGAIFPIKAFLLMGK SYFAGEFKLVHMFELSYPWIFEDYKNTPVIFVMMFLLTL FFDVYSISMYIGYDPLVPIFMLHLCGQINILKIRISQVFSD PSDSDEIVRAKIRIIILKLQDIYSFIEVIKTNFTMLYEFMM KTTTFLLPLTAFQVTESLRNGEVNLEFIGFFTGVILHFFM PCYYSDLLMETGNDFRLALYSCGWEKHSDLRTRRTILF MMARATKPLVISTVFYAICLDTFAKMCREAYSIFNLMN AAWA	-	-
PoctOR6	KM892369	1248	MDVFDSRYMKNIRFTLRSIGAWPSHVFEDLPTTKLSAIS RYGYTCLLFTFCSTGMVAQAVYLVKNKGTMPFIDLGQT YLTLLMSFAFVQRTTMPIQPSYQALIKEFVSKFHLLHHK DKTDFSAKMFSKVNRMCEIGTIVELLQFGVSASMFNIAP LALNYSRGMLSSERPMNVTFEYSVYYALPVDQNTGVW YIVICMYNAYVSYNLGCMFLCHDLQISVFVFHIWGHMN IFEHNLNYFPRPRKAVGSKSDPLRYSNEESKEVTMGLKE IIKHYIMIKDFVAKTSDVYSVTLCVYYGLHLVADCVLLL ECSTLEIDALAKYGLLTLVMYQQLIQVSVVFELISSKGES LPDAVYGLPWECMDNGNRRTVLILLQIVQQSLALKACG MVSVGLQTMLAILKTSFSYFLMLKTFANN	-	-
PoctOR7	KM892370	1248	MDAFNLRYMKRIRFTLRTIGAWPNHEFGDHPATALSPIR RYGYTTFLFTICCIGMVAQAMYLVNNRDTLSFVDSGQT LVTFFLCVVYIQRTLLPISTSYQAVIKEFSLNFNIVHHED KSDFSAKMSSRISKICEILCTVIHLQIYIAPFFFNVVPLIIN WNNGMFGPDRPVNATFDQSVSYALPFNQRTYYSYVIIV	Male	Male

			VYNYYVSYNLSCVFCIQDLLIYVIVIHIWGHLKIFEHTLN TFPRPKVESKSGTNLARFTEEENKEMHYKLKEIILTYLNI RKFIETTSTAYSETILVYYGYHMIADCVLLLECSSMDPK ALLRYGPLTLVNFQQLIELSVVFELISSRSEGIINSAYDLP WECMDNSNSKMVLILLLIVQESVAVKACGMVPIGVQT MVAVLKASFSYFMMLSAFADD		
PoctOR9	KM892371	1185	MKNYYILKNLCGKIYLAGAGNFWYEEGEIGNDKSIKYV VLSTIMFFIYVCMTILEIIAAIFSDLPKDEKSDSVSFAMSH TIVLIKIFSVMGNRKLVKEVNYKMVKICEAYEDEKRVA ENYKIMKMNVYAYVAAVYGSLACFVFEGIRKMQSGSH FITIVTYWPFFEDESLSAVLFRFFTTCVLCVLMVTMIAID SFVMVTLIMYKYKFITLRLYLENLREEFDKNDYAGNEK DAAGKLQSGLIEGIVMHRDLIRLSNDIDRSFGTVMALQV CLSSGSAVSLLLQIALSKDLTFVAGMKIIFFVIALFFLLAL FLCNAGEITYQASLLSDSIFYCGWHACPMGRDLRRLVL MACASAQRPLVMKAFKMLQLTYGTFLTVVRSTYSVFA LFYAQNE	-	-
PoctOR10	KM892372	1224	MMIVQKIISFAKRLEDPKYPLLGPNLKGLYVFGLWQSGS KLRNSCYNFIHFSAFLFVFSQIVELWIIRNDYLEALHNLS LTALGMVCIFKAITYVLWQSDWKELVECVSAEEIAQIG KQNETCAKLMRNYTIYARIVTYLYWNVTISTNITMVSA PFVKYATSPEYREQIRNGTEPLPQIFSSWFPFDKSEVPGY FVPIFVHILVNIHGGGMIALYDSNAVAVMVFIRGQLAIL RENCKNLFDDYEQVTRREILDRIKEFHRHHNFIVKQSAL FDSLLSPVMFLYILVCSGMICCSVIQFTSDEATAAQKIW VLQYTAALIAQLFLYCWHSNEVIVECNDVDGGLYESD WWKGDVRVRKQLVILGGQLRHAVSFSAGPFTSLCVPTF IDVIKGSYSFFTLLTQMQEE	Female	-
PoctOR12	KM892373	1179	MPPPQSDSFRHNCFFWKVFGIWPGRTPNKYYKYFSFTY LITTLVAYNALLTLNLYYTPRKIDLFIHEVIFYFTEIAVTT KVAMILLKRKQIVGIFELLDYEEFKGSDDTGRKIVETHN SNFKIYYKIXSILXNFTYLSQVIYPVIGMLIFKTTLDLPIC	-	-

			KYYFLSEETIDNFFTLIFIYQSFGMYGHMMYNVNIDTLIV GFMVLAIAQMKVLSHDLENLKVEKTDTGRGEELDQKQ LQKLYKCVKHYALVLKYCADFQQIISATMFVQYGVGA ACICVTMCALLLPLKVETFIFMVGYFFVMSLQLFVPAFL GTQLLYESQELMVAAYKSDWIPRSRGYRQSIKLFVERA KHPVVITGLKIFPLSLATYISIMKTAYSCFTLVRFMQDRE EH		
PoctOR14	KM892374	1113	MIVGMLYPTPGTDWRRAIGIAVAMASVVPISGMAALDV WNSWQRKDIVNITRHCTVIGPLFGVLFKMIIFFINRAEVY RIIKTIDGDHAKYNTLPERYKAVARALLSDSMYYSEYL WACVVGFCVLTFPLSAACLNVYNCLCKAEPTLYLVHD VEKPFSRPEERFDSPFFEIMFIYMTYVSVLYIFNFTGFDAF FGLTINHVCMKIELLCMSLDDAMLETDPKVKFEKMVG VIQEQNNVYKMLELLQETFNIWLGIILIATMAQICNCMY QVLEGYGIDPRYLVFIWGTVVHIYMPCRYAAKLQAAA ADIATRAYCSGWERSGDLRSRRMVAFMIARAQVPVNIT AFNMINFDMELFVSLLQKSYSIFTLL	Female	-
PoctOR18	KM892375	1185	MSEKNVALFLNRPRNILGFFGIWLPPASFTLLYTIYMLFI LLTQYSFVLFEFIYIVEVWGDLDAVSEASFLLFTQASVCF KVTRFLIHKDKLVMLLEFMEEEVFQPQNKIHERYLITLS TRVKRLCLFFLMSACTTCTLWGMVPVVDSTAGERFFPF LIWMPVGPANSPQFELGYFYQMAAIYISAFLFIAVDSVV LSMIMFGCAELEIIMNKVEQIQKVPLSGKLKQDEREQRI RDNCELFKKCIKHHQEVIRFIDIVEDTFHANIFFQLSGSV AIICIIGLRISIVEPGSMQFISMLNYMMTMLSQLFLYCWC GNELTIRSELLRDVMYLSPWYEQSNPFRRLLWVAMER MKKPILFKAGHYIPLSRPTFVSILRSSYSYFAVLNQAQNK EK	-	-
PoctOR19	KM892376	1197	MESQNSDKKISPKYKDFNETFKFCSFALAIGLIYPNKRN VRKRVILFLYVLLFNTFTLFWMFWYTFKCLVNHDVYNS TRNITLGVIVFLFIFKTCYVNMKTAMFSEVLKQITKDLL KGNEMEEDYQEIYDHFIKQGKLGQSVYVLIPCMLSFMF	-	-

			PIYSGGAMIGGSLKNDNFTRYMIHEMDLKYVEDKQYHSPYFEIIFAYFTTPCFVLLPNYVGFDGSFCIATSHLCLKLKLMAHSIKKAFEDSKSTTELKARLKICIKDHQEALEFHVLIQKTYGGWLFAVFVLTSFLISCNLYQIYLVGIDPKYTMFAITGVFHMYAPCHFASNLIAVGDEVCSDVYCVKWEAWGDPSITRLLVFVMARAQKNVQLTGLNIVTYNMDTFVSLMQTSYSFFTLITR		
PoctOR21	KM892377	1245	MEVFELRYIKRIRFTLRYLGAWPTHVFEDCVTTKLSVIRRYGYTMLLCVVCSLGMVFQVQYLIKYKGILGFIDLGQTCLTLLICCVFCQRTTLLTQRTYRELIKDFVLTFHLTHHRNNSEFSLKMCNRVNKICEIATIVQLVQIYITALMFNIVPLYNNYKAGMFSSDRPVNGTFELSTYYDLPFDQNTDLWYTILCIYNFYASFNVSCMFCCHDLLICVFVFHIWGHLTIFEHKLNYFPRPRKAVGSKTDPLRYSFEENKEVANGLREIIKQYLMIKEFLANTSATYSVTLCVYYGFHLVSDCILLLECSTLDADALAKYGMVTLVVFQQMIQLSVVFELISSKGDTLVDAVYGLPWECMDNGNRRTVLILQQIVQQSLALKACNMVPVGVQTMVAVLKASYSYFMMLNTFAN	-	-
PoctOR22	KM892378	1230	MTVEKDPNFEYYLKPPREQVFYKGMAIAMTFLRLANSTWWGLPPTLKLLKIHGDMFLIVAPICLTLQVVYLYVVYFSELSFTTLGTMFSMIPATLVVNVKLIVSMRSAYKQIMYNLMDKIHIYNFLDDNDDFIKTKLLKVERNTRWLTTFLVGFILMDWLLWSVIPLRNNIINKERIGNRTIRLETCLYMWMPFDYGYEFRTWLITHAMNVYLVFTGGLVLSFCDSINFIFIFHFLCHVDILRHKMRTYFAVKLNDTDTKGKIVEIIKYHSFILSTFKEMEAGFGANVTLNYLQNLVNDSLLLYQIMVGDKANRFTYLCMMQFHMGGLILISFALEQIRIKTEDLALDLYQVPWEKMSTSNQKLLIPILLRMQTPLVFEGALGLQTGVRPLASIIKSTFSYYVMLKSSIE	Female	-
PoctOR25	KM892379	1131	MFGNLFELSSGIFAFNLKYLFIAGLWPSDKWAKENLFIYKIYEVSLHILGATFLIVTGIGTYRSKNDMMILLGNIDKSLAGYNLLAKVIIFLYKRKELENLISEISRSGDQISEERKKL	Female	Female

			MLIHVVVLTGMIMSIAGLFSGLAFYNNEMTIEAWMPFD PMDSKMNLMLSMQILIITFTVPCVWRGIAMQGVVVSLT MYVCDQLVDVQERLRALVYTPDSEFAMRAQFKDIIKK HIRLVGYSRTMTDIFNEYFLIQNLAVTVELCLSAFMATI AGLEQQSLLASFLAFLCVALTNAYIYCYLGDELIVQSQG IALAAYESTWTSWPLDLQKDLLILIRAAQKPLILSAGGIA AMSMQTYSQTLYNGYSIFAVLNDAEIK		
PoctOR26	KM892380	1185	MSEKEGVPLSYLSLVPHLKNLRYCGILPLPSDNPILKRRL HKIYMGFSFSFIMLYTVQQIVNVYQVRSDVDKVMDSMF LLLTFCDSICKQVAFIMNPHKIQEMLEIMQGPNFNQGRE EHRPLLLKTVFQAQLLLKIFNTLCLLTCLLWVLFPIISHIK EEAIEFTIWLPFDANQNPQFYVAMVYVWLQTSWLGYN NSTMDIFIMYLFAQTKTQLTILRIDLENLVSRSKTEAAVS SFTYTDLLEKHFRGIIHHYNEIIKFSSMNEGIFNGPILFQF LISGWIICTTAYRTVNMSPASGEFSSMILYMICILSELFLF CFYGNEVAYESEILMESAYCMDWTEMPVKYRRFLIIFM ERIKNPIKPKAGKIVPLSINTFVQVVKSSYTFFTFLNKA	-	-
PoctOR27	KM892381	1209	MLKKYVSRLQDPNHPLLGPTLWGLQSWGMWQPNSGL SRIIYNLIHLAAILFVLSQYVELWLIRSDRESALRNLSVT MLSSVCVVKAGTFVSWQRHWRDVINFVSTLERSQLKK TDKTTHNVIEGYTKYSRSVTYFYWSLVTATVFTVILAPL GAYLSSAEQRELMGNGTIPYPEIMSSWFPFDKTKGVGY WIAIVEHSLICFYGGGIVANYDANAVALMSFFCGQLELL SANCKAIFGDGSGLVSYSEAMNRIKECHLHHLSLIKYSK ILNSLLSPVLFIYVIICSLMICASASQLTNQSTTTMQRIWV AEYLVALIAQLFLYCWHGNKVFFMSERVDRGVYESEW WQCGVRLRRCVLLLGGQLRRTILFAAGPFSTLTVATFV AILKGSYSYYTLLSNNED	Female	-
PoctOR29	KM892382	1197	MIKSFLGSLEDPNRPLFGPNYWLLNKIGLILPDNTWNRI YYVLIHEIVAFFVFTQYMELYVIRSNQELVLSNLKISMLS IVCVAKSNTFVIWQNNWRQVIDYITKADIFERDNQDKA KGKIINSYTKYCRRVTYFYWVLVFTTFITVITTPLLIWYS	-	-

			SYDFREGYKNGTEAFPHIFSSWMPIDKENSPGSWITVIW HTALCAYGATIMAAYDTSIMVILVYFGGKLDLLRIRCKE MLGVIEEAISDKEAEAAVSQLHGIHVLLLKHSRMFNSVL SPVMFVYVVMCSLMICASAFQLTSATNSTQKILTAEYLI FGIAQLFMFCWHSNDVIHKSQKVTMGPYESQWWAVGL RQRKSVLILQGQLQIMHIYSAGPFTDLTLATFVTILKGA YSYYTLLRD		
PoctOR30	KM892383	1188	MAILKFDDLYALPVTCLRLIRAHPSIPRDRSWVWQVAL MHGLFSVGFSSVVYSLIAVDIKANDFIGTCSNGIFVVVW VVITFCYGVILKFSDELNTLMRLNQGYFDAADSLPEEDQ AVIREYAFRARWVARLWGFACGLNGLLFPLKSLVLTLY SIFIDDFRLVPMYEVSFPPAIENIKETPVMFYVIWVMFAI YAAYASIMYMGFCSLGPIFIINTCAQLELMAKEFKNMFN ITPYDYKLVGVKLSAVIKKSQDIKANVQIINDVFRVYYE FTLKAAAIMIPITLYLVIDTIKQGELRIEFLMFDITATMLC YIPCYYSDLLMEKGEAVRLAMYTSGWENNFDKKARAS LLIMLTVAARPISISTIFRVVCLDAFAGMCHEAYAIFSLIN AMWN	Male	Male
PoctOR32	KM892384	1260	MKKSIGLQLHRYLPVFNSGPPKLVNTIQRLVWNTGLWS NQSLGLHWLAKLAIVCFFSTNFTQFAAMIIERDDSERVF ECFSVLSFCGMGALKLVNLYFNRERWLFMLSQVTLLEN KQLDDASDKQSVEYDSDDEEECLSPHIERYTRKHTVTS NLLLMLYSATAVIFIVTPFVEYALQKNPKAYPHILPGWA PLDAVGFLGYFVSVLFEVVGSIYCVFVHVAFDCTSVGIM IFICGQFSMLRYSTENIGGRGQDCRLSAARDARARLRIA KTHGTHIILCNVINELGASLRNILGVYFLVATLTVCSVA VRINSQTLSFMQLVSLLQYMCGTLTQLFLFCRYGDAVF HESYFSMGQGPFGAAWWGLRPRVRRELAMLGAAMAR PRALRAGPFNTLDLPSFVQIVRAAYSYYAVLGQTSK	-	-
PoctOR35	KM892385	1233	MEMSNCHRDEMTRTRKKEMSRFDIRNIFKFLEDPEHPS VGPHLHLLRLTGQWHPNDSIPIRLKLVVFFVIVLLFSSQY VKCFMGIEINSLLLILQYAPFHMGIIKTIFFQKDYSKWKE	-	-

			LIGYMSSTERRQLNEDKSEAEQVMDSYIKRGRRVTYFF WALAFFSNFTIFSEPYQKNQTVENGTDVYVKIFNFFTPF NQDVPPGYYVSMAIQTVLGHIVSAYVVAWDTIVVSTMI FFAGQLKISRIYCVRVIETGSAEQSHRNIVMCHRFHGSL VKYQKLFNSLISSVMFVYLVVVSVNLGVCIIQLSQLKDD LAALVASLVFVVACLIQLLIFYWYGSQVTEESVLVSYGI FESDWFKTDAKLLKEVLLLQLATSKRLVFRAGPFNEMS LTTFVAILRSSYSFYTLLNETK		
PoctOR37	KM892386	1170	MEHLNNLKESMAVTKARLKENSLDSLVWLGNIVPRILG FDSAREKVFIPYWILHFSLFTYVYVVGCVVYQARHAKV ASDFVKNYVNVSLVILTVMNSYWFLVERGLLKTVLKK VKEIDNYVVQSGLFAEKHTHSLFLIKRIIHIFGGLNLISFF VVNIPNRLDLKIETFSMAICVGIEPLTSSPNREICKVLLG MTECSLGAVTLHFQTLMLILIAHTTSMYQILSDDIMMFN TELEDHRDYAYIKDKLSMMVYRHALILEITQGLKSLYS MPMGVNFGLNAVCMCFFCFLTSDEYLGFMPILVYSFLA FFLYCLLGQRLTNAAEVFARAVYSCGWEKMNVKEQKA IYIMLMQAQKPVELLAADIIPVNMYTFASTIEAIYKFTTA VKL	-	-
PoctOR38	KM892387	1329	METLRKFGLKHHDFPTMMWNVAVLLRALALDVDGRN TTGISPLNYLFMLVTFVFYFYTYHLSTIWFVFWRADGDL LFYILLLSLSISSLIGVIKLLYMYVSSKKLRQIVADYLLCD AAIIPGSRLANNIKVTLRKVKRRAAIYWLIIIGNGVVYVV VPLLTPGRHITVDEQILWGLDPMFESPNYEIATTILWFAV FLTVYAPANITGFLIIIVGYSEAQMMALSEELLHLWDDA QNHCQRLPEDDNLPMEASSSSGTNFFARRSKDSIILSGPA FKENETTAPETSVQEKTINGFIKQRLVSIMKMHAVNINLI KRVENIFQNAMALEFTLLSGGLIAELLGGLENTYVEVPF ALVQVAMDCITGQKLIDANAVFADAVYGCKWENFDLS NMKLALVMLQNAQKTMKLSAGGVNSLNYSTLMSVIKS IYSAYTALRSTMNT	-	-

PoctOR39	KM892388	1188	MISNVAMMLRVVALRIDPRNKKPIPTFLYILTSVCVVCYIYNYQISMVWFVFSRCQETGDVVAAMMVLSTGTTSLIGITKLFTVYYYQENVQSMVGRYIEYDTTMIAPASSALMLATMRNVKKRALLFGVVMMGNGFAYNLQPLFKSGRHLMDDDQILYGLEPMLETPNYQIALVNIIFSVNFICFIVSNITGLLIITTGYSESRLLALSLEIQDVWQNALQHYRDNFDANYTEDKYDETATVELNKYVKHRLFHIQKSHFTNINLVKSIEDIFRQAIVVEFFLLTLSLIADLLGGLENTFIIMPFALMQVGMDCFTGQRLMDASFVFEAAVYDCKWENFDASNRRTVQLMLQISQKTLILSAGGVTALNFACLMSMIRSIYSAYTALRSSMNYK	Female	-
PoctOR42	KM892389	1140	PTFAEVFKQIKTNFSLMGIPRDKSMFNVRFYILIAFLIVMLIEEIFFFVKKMAPENFLELTGLAPCICVGCLSVLKIIPVACHRTTVFSLAGKLSELSTEILKDPKNEKVIKGNINSVRTLIKYYVILNLVLISVYNFSTPFYILYHYIASREEIFFLPYPVLVPFSTETWVNWFIVYLHSICSGFICVLYFTTVDGLYFVLTSYVCSMFEVLSKDIKEIDNDTTDLKEIVKRHQYVLILAEDLEVIFTLPNFFNVLVGSIEICALGLNLMIGEWGDVPGCILFLSSVLVQIFMISVFGEKLITESTKVSDSAFLCKWYEMNAKSKKTILMLMIRAHKPQRLTANKFSVICFNGFSKIISNSWSYFTILWTVYSRKNEQ	-	-
PoctOR44	KM892390	1185	YHAVHIYWLKFYGLWYNTVSKCSFQFWTHIVYGFLVLWLVCFLPGIGEVVYLLRRRDNIGEIAEGLYLFLSEMYTYIKLSALWLNKKKIIDLLEYLHREEFKASEVEHREILRKSIKTARFVMTYYSTICVGAVSVAIIMPLAEDFEVLPTNVEYPYFDVYKSPAYGIIYIHHIYYKPATCIIDGVMDTILSAFIASAIGQIEILAFNLRNFNLLAERRQKRSLMGDINNEESTRYHVRAVFKDIIKHHNSIIRYVSLIESAFSLASALQLMLSVMVLCLVGIQFLSIEEPMSHPIQIAWMAIYLTCMLIEVFIICWFGDELIWKSKELRQAAFDAPWPTTDPKTAMFIIIFMERCKRPLRVTAGKIFTLSLDTYTNLINWAYKAFAVMRKMKK	-	-

PoctOR45	KM892391	1248	MDAFDSSYLKRIRFSLRSVGAWPNHVFEDLPTSKLAAIS RYGYTSFLCTLCCIGVVAQAMYFVNNKGILPFIDLGQSC VTLLLCLVYIQRTTLPIQTAYQETIKEFVLKFNLIHHKHT TDFSAKMYDKVNKICEITSIVAHIQFYMSAFMFNLAPLC LNYRAGMFSSERPVNVTFDHSVYYALPFDQNNGIWFTII TVHNAYVSYNLACNFACHDLLICLFVFHIWGHLTIVEH NLNCFPRPRIHVGSKIVPLRYSNEENKEVAAELKEIIKYY IMIKQFITKTSDTYSVTLCVYLGFHLVSDCILLLECSTLE VEALAKYGVLTLVIYQLLIQLSVVFELISSKSDCLPDAV YGVPWECMDDGNRRSVLILLQIVQQPMPLKACGMVPIG VQTMLAILKTSFSYFLMLRTFANN	-	-
PoctOR46	KM892392	624	ITINYTCFLSCDRETRLQQLLYFCLTTVTVAGWAGSAEK NQLPLRAWYPYDTSKSPAYELTYVHQVGALFIAAYLNV GKDTLVTGLIAQCRCRLRLLGLGLRTLCEDLVPNEQGIL TPEQEKTAWSRLRVIVQRHQAALEASSLLQDCFSAPIFA QFMVSMVIICVTAFQLAAQTGNLVRLFSMGTYLLNMTF QVFLYCYQGNQLSEE	-	-
PoctOR47	KM892393	1362	MPDSKAADRPTRYFATHFYLLRFLGLGWWHHPDEDNT SNFPGLYIYYTIITEIVWVAGFVGLETIDPFIGEKDIDRFM FSLSFVITHDLTIIKLYIFLFENRAIQDIVRTLEIDIYNFYQ NNERNRATIRITKIMTGSFVFFGWITIGNTNVYGTIQDIR WKAEVALLNDTSLRPLRTLPQPIYIPWKYQTDVSYISTF VLETVGLLWTGHIVMAIDTFIGSVILHMSSQFVILQEAF MTSYDRALKQLLTEIPDSMDARENIIMPNEDDDNTRKD SLDKIEAKVKSFYTDEQIESAVEKSVLNCIRQHQVLISCV EKFRVTYSYGFMTQLLSSMAAICVVMVQVSQDASSFKS IRLVTSLAFFVAMIIQLALQCFTGNELTLQTERVSDAVM QSKWERMTPRVRRYLLLAMLRAQRPLRLTAAGFAYMD NGCFLAIIKAAYSYYAVLSQKEV	Female	-
PoctOR48	KM892394	1194	MFREFLDRLRTEEMPLLGPNTWFLKTIGLRLPKSKIKKV FCVILHEIVTFFVITQYIELFSVTDDVDTMITNLKSSSLSII CVIKSNTMLLWQDKWLEAFEYVTEADKFERVTGNPAR	-	-

			DKIVTRYTKQCSLVTYYYYLLLFFTNFGVISLNLFVNMT NVEFRAALNNGTVPFPHIFSAWTPYDRNTFPFTWITVV WHVFITTTGAVIISAYDTAAIVLMTFFGAKFELLRLRCA ELFQEGEPVTEEEFDERVRQVHTLHTQLVKNIRLVDSLL SPIMCLYTVACSLVLCTSFYQLSFSTSISQKLMLAEYLAF GITQLFMYCWISNDVLEKSSKLMLGPYESQWWAGSPR QRRTVLMLAEQMEKAHVFSAGPFTNLTLPTFIMIVKGA YSYFTLLRN		
PoctOR51	KM892395	1173	MADILKEIHPQKYYLRLVSRLMFCFGFGTYWYEGEDTR YPRLYTVWCIIIQSYIFLVVLDLILAILRPDLSDEEKSAVI QVGFSQLLVILKFIIVVLQRHRIRDGFKKLLEEDRDIFTSL ELEKESVKTAKVFFLPFMIASYSYLTLNLVWSTFVSIMH GTPIQTQITYFPTPALTGFPYNFLRILIIAHWWTHATMMI TADCLCSLPIFFVTAKFKQVQMYFEILGENNLEDWSGEE FRKGFVNGIKLHQNALWCASNIQSALGILYGVQIWQTVI LIGITLFQLASVERTMSNMITGLVFIVCVSLLTGAYMINS GIITYEAAKVATSMFHCGWERTRADRRLRTLLVVAVQR AQRPVYMTAFGMMNLSHESFVTVLRAAYSFFALVY	-	-
PoctOR53	KM892396	1140	MQPSDCFKISFFVLIVAGVWYPPSWEKSRYLHAVNIYR VFAMIMILIGMLSIQFVYFFTVVGVDLDKTIDATALFTFV GHLYKAITVIINRRRINKLLEIIDSEEDKDDLMQNMAAKI NLVAKIYNGGAGLTAVMWNLIPFTKPTLTLPFYYPDLPS DSPWFVRWYAYQTVILIINGVAQTSADHVFGGLMAFAA TQLKLLQYKLETIGSRKETEELDAAQREQDDYKEAVSC VRFHLKIIGVVDELTSIFGAAAFGQFVLAAPLLCLSAFIL TTSSDPTEIITRLLYFGCISGQLFFYCFCGNMIKIQSDLVA TAAYDCSWETTSVRTQKTLKLLILRGQKTLSVVAGNLF ELSLVTFGALLKSSYSFFAVLNKKHND	-	-
PoctOR54	KM892397	1131	MKNSQCLSSSIAVMKYSGVWKPDGLTYRWEMAYKIFG FVTQVLFFYFIILAEIGYVYTFLDNVERMVDAAVLLLSH LVQAVKVMTIILRQGRIKRMIEMVDGPAFTKSDPKLKAI LESSTRLAAMVGNVILCSACTTGIFWGIVPALNPELTLPL	-	-

			RTAYPFDIHGPYIFPAMYAYSSISVIVVGVGDAAENFLV<br>SGLFTLASAQVDVLREELKAIGADGCTDNFEKATLCVK<br>YHQRIIGYVEEMADIFGLPMFCQFVTTSVVICMTIYKITV<br>TQEPVEMVTMVFYLMCVFMELLMYCYPGDVLLNKSLL<br>VSEAAYPNDWSNDKRTSRVLLLTALRAQRPLAIDAGG<br>MFRVCLPTAAAVVQTSYSYYALVRQQLAKE		
PoctOR57	KM892398	1008	MYPSFQAFREDFDALALAGYFKIVSKPVSRLKRSLHDA<br>YRTAVWVVVITYNLQHVIRVIQARHSTEQMVNTLFVLL<br>TTLNTLGKQIAFNARSAXIDRLVEIIEGPLFASRNAYHEE<br>IMKAHALEMSRLLKLYHAAIYMCGVMFGVFPLVNRILG<br>EEVEFTGYFPFHTNDVLPFALALSFNTIVITFQAYGNVTL<br>DCTIVAFFAHSKIQLQMLRYNLEHLVDRNWTKLDTKIN<br>NTSVPKTFIDIEDAAFGELLRKRVAHCVEHYKIIVWFTN<br>EVETVFGESMVVQFFVMAWVICMTVYKIAGLSLLSAEL<br>VTMALYLCCMLAQLFIYCFYGTQVKYE	-	-
PoctOR58	KM892399	1209	MELPDTYCTNKTVYLFRKICKVIFLSCATNFGFEDTNLP<br>SCFIRIYGLISKILEVIVFVFLASEWGSFYTQKNLTEKQLS<br>DMYLFAFSHVVLYAIYASALYHRERIRELVLALTVTLK<br>QVCNDEATERVMIMKTYRSLTAMVFVCSGSLFSYGLD<br>AGVQAFTSNATFTTVIPVWPDVEDRQFIASVARIIYYIIW<br>WIFVVRVISIYLIVLCITICLGHQFTNLHKYFLKLNDIFEE<br>NGSQEDKERRYEKAVKVGVKMHSITLWCAEQTQVTCG<br>VAYSGQVIINVSVLVLLMIQMVHTERTLTSAAPIAMLGA<br>SVLVSSAVFMLNAGDITIEASRLPTAMFLSGWHNCRGQ<br>ASVRARKLLTIAMAEAQNPVVIIGLGVIQLSYQSYVSIV<br>KSSYSLFSVIYSY	-	-
PoctOR59	KM892400	1179	EARGEINETLSLCLFCMRWIGLSFEPPTSTRAYLRQKLM<br>FAVSVCAIVYHVFSEIVYIGLTLSNSPRVEDVVPLFHTFG<br>YGALSIAKVFALWYKKDVFAEHLQELSGIWPMPPLDEN<br>AQRIKETSIAALRLVHRWYFAMNMGGVLFYNVTPICVY<br>LYELWSGQDAVVGFVWVSWYPFDKYQPINHVFVYIFE<br>VFAGQTCVWIMLCTDLLFSGLASHIALLLRLLHSRLETL	-	-

			AETKKTNEEYYQEIIGNIKLHQRLIRYCNDLEEAFTIVNL VNVVLSSVNICCVVFVIVLLEPFVAVSNKLFLGAALIQIG MLCWYADDIFHSNADVALAVYKSGWYRTDPRCRRALI FLIRRAQKPIAFTAMKFTNLSLVTYSSILTRSYSYFALLY TMYN		
PoctOR60	KM892401	1218	MSETHDTSRKEIHKSLDLLNFCTRMIGLSFIDDPPTSVFA KIKSSFIFGLSALSLMLLFTVEVSYIVSLVIKTASLADLVT GLHIVGYGVMSTSKLLTLWVKRHTFRKSIKDLAEIWPE APENQEEEDLKKGSLKALRIGQFSYTFFNVLGVVIYNITP IGIHLYRTSRGLPSVMGYVWFAAYPFDKTKPINHAFVY AFECFSGACSMWSMVSTDMMFTTMASHISLLLRVLQIK IRRLGFADAPTPTDNARSYQEIVNLIKIHQRLISFSDNVE DAFSLVNFINVMISSVNICCVMFVIVLLEPWVEMSNKFF LVAALIQVGILCWYADEIYQASVGVADAVYASDWYYG DVRARRALAVFIQRSQKPLYFTALKFRPITMITFSSILTTS YSYFTLLYTVYSD	-	-
PoctOR61	KM892402	1224	MSCHDEENFQNDIDYVATVASRICLYPFYGRPKYKIFCY YSICFLIFFVSAQQFTALCVYGFKSFLDIVGIAPNIGVTLM AVTKYIKVHRNKHTYNLIFNHLRSNMWDVVDKSSQEN RKILITYQKVVKFIILWYVYYIVPLLSIIVTFPLLIMYYDW KVLGKDLELRYPFEAWYPFDKIKWYYAAYTWECVMT AIVVYIYTYSDTINVSYIGYICMELKLLGTHLRHLIGTKEI MDLKNSHEVAAVHDKIKCKLRRIILKHVFLANIVSQLD GILGDIMLVNYTLGSIVICLTAYTFTVVDEFYSTVRFFFF FISFIISILNQCVMGQVISDHSEGLAEELYNSEWTYGDRD TKQLVLILIMRMQKPFQLTAKKYIAMNLHTFTAICSTSY QCFNLLRTMYDPKKNK	Female	-
PoctOR62	KM892403	1068	MTLFGAGETWYLVSNYHLLDIFIEQLNVMVIQWTAIIRF KSMRRHKDIYKKLAAAMESSNFDTSTPARLALVEYWR LRSEKYLKVIYGLGTCTLAAWFVYPLIDDVECNLMVAV RLPLDFCSPVRYPVAFLMTMVAFCYVAYYVMTNDVIM QTHLMHLLCQYAVLIDCFENILEDSERNFKGVSRNDLIH	-	-

			NNDFRITYLRRLGRLVDQHKLLLKHTMDLRQSLSAPMLAQVMASGMQMCFAGFQVLMTITDSITKFLMSFLFLGYNMFQLFVLCRWCDEIKTQSTKIGDALYCSGWERGLTTIPGVRRRLLLVAMRANKPLVLTAGGLYDLSLSSFADLVKTSYTALTVLLRLRHD		
PoctOR63	KM892404	1182	METAFFESAYRVIYITGWSSCDRGIGYQLYCNSIKLLMALFVVGETWYLASNYRSLDVFVEQLNVMVIQFTAVLRFKSMRDHEHIYKRLAATMESPTFDTRTRARQALVEYWRLRSERYLKLVLGLGTCTLAAWYVYPLIDDVECNLPVAVTVPVDYCTPVLYPVVYLTTTIAFNYAAYFIMTNDVIMQAHLMHLLCQYTVLVDCFENILIDCEENFTGVNWSSLIHNNNFRLKYLERLGRLVDQHKLILNHTMELRKVLSSPMLAQVTASGLQICFAGYQVAMTITDSFTKFLMCFLFLGYNMFQLFVFCRWCDEIKSQSAKIGDALYCSGWERGLTTIPGVRRRLLLVAMRANKPLVLTAGGLYDLSLSSFANLVKTSYSALTVLLRLRHD	-	-
PoctOR64	KM892405	1290	MTIVNNVKSFVNKEGFDWDRPDMTLQIFHPQLEMVFAANGIFFNNRESKIRFIWPVLCALLSLVANSFEIMFIWHGVAIKDYGFATESFCYFFILGTIIVVYFSMLTNREKIFLLADNLNKDFLYICNLGAHYRDTFLKGQLLIWKLCWAWLSFALFIMILYVGNTLIALLYQSTLATQDEHMVRPFMFPIWLPADDPHRSPNYEIFMSLEIILIFVVFCSFIFYVYILFHLLLHYYNLMDMILIAFGELFDDLDESVVTLPTEDPRRKAVQAELNRRMGQIARWHHSVFESVETISSVYGPALVYQTMFSSVVICLIAFQVAEQLSEGKFDYLFGILGVGACMQLWIPCYIGTLLRNKGFSIGDRCFYCGWHSTPLGRLLRRDFVIFIQRSQAPLAIKFTALPHLQLETFSSIMSSAYSYFNMLRQYN	-	-
PoctOR66	KM892406	1203	MTKYPKYKKSKATDFFFNLNRVIFFCNSLNFWVEEVGVSALFVKFYQSLSKLINIAAYIFVAFEWGAFYTQNNLTEKQKGDRFLMSFSHTILYSFTVIMIHHRERVRELWFTLAVTLKKDFNDAETERLMIKTTKFYTSAFVVICGNALIFYGIDGIIQLAYSDGTFVTLIPFWPDVNDHRTVASAARIATYVF	Female	-

			WWLFMARITSVYLLVLTITICLSHQYTNLQLYFKSLENIF<br>KQNIPQSIKEARYERALKIGVKLHATTIWCTQQVQETCS<br>MVFSGQIIVNTTVMVLLLSQMVASERTLGNTLPIAATIV<br>SMLFSTGLIMWNAGDVTVEAARLPTAMFLSGWQHCQD<br>KASYRIRRLLLIAITQSQKPVVIRTLGVIELSYQSYLPIVK<br>TSYSIFSVLY		
PoctOR67	KM892407	1191	RRKMRSLLLVICYVTFYVSLTVSFSKVFTGVLGFYDLAN<br>LLPIFIVATQGAMKGAVIITNLSKARTLIDELGAMWRTS<br>GLTRNQLARKGMMLKRLNLCNAVFYWMNIVGTWQYI<br>LVPLFETLFRTFVLGQDKQLFPFICTFPFDPMRNWLVYL<br>LTYFYESYSMLHLIYMYLGVEFLMITLCSHLATEFELLR<br>EELLHARSRKETIDVLDYVNCSGDIENGDSIVEHDTIDID<br>DGIMLDEDRPDIKDVIRRHQKLIMLSELLDDIFNKMIFFN<br>LLFATITICFFGFVAKIARDLPEMANNFVGVIASMIPIFNL<br>CYYAEMLSGASAGVADSAYHNLWYEGDLRYQRIIIFIIV<br>RSQKACSLTSMRYSPVTLNTFTTVLSTTWSYFSLAISVY<br>ETDKQ	-	-
PoctOR68	KM892408	1206	MPPTLISTFSLHLKIFKIIGLDFLGDPSVANHCRHVSFVS<br>MLLLFFTGQLLFFFKSDEIGADFMDIANAIPLFMMAVQD<br>LVKIVALSKMQRIKGIIMEVAELWPNEINNEEKKSIMNS<br>WIWNLKMFNDCVYKFVAFAVFVFVWGTFFVTVFTTSD<br>GVITYLYSFQLYYPFKIDSMWKYSAAFLFQSVTGTIIHLC<br>LYQPCDLLLFTLTVDICILMRLLQYDLENIRVVGKDRNG<br>VFDPAEAEKSYRAVIELARTHQKLVKISENLNEVFGTIIF<br>TVVSLSAVILCFFGFLLITVGGTQYQMLRSFLAVFVKMF<br>IVFCLALPGQILSDASCGVADAAYKSLWYESDLKFRKIIF<br>IMIARSQKPCFLSALGYSQMNFNTFCKICSSSWSYLSLLN<br>QMYQDTER	-	-
PoctOR71	KM892409	1332	MAVDSKPIEISDYTHFIIISLQIVGCWDWFPNPDKQYKIII<br>NNIYLALVLFVLINFPTTLIVNLYTEWDNVMGSLEMLA<br>DGLPLLVAVAVVIYFALYKKELYELVEFMNGNFKFHSA<br>RGLTNMTMEHSYKSAKNFGFVYTACTLFSVTMYVMLP	Female	-

			MIVHLWTKQPLQNWMYTDIVQTPFIVMAFLYQCLAQA FVGLAVGQLGVFFAANAILLCGQLDLLCCSLRNARYTG LLQTGVQHKVLLQEYAGIVDDERHNYIYSETEAKDSEY HYDAKVTSYFVDRRSEFDIYSAEFDAATAEALRECARV CQVVATYKDLFEDFVSPLLVLRVVQVTLYLCTLLYAAS VKFELTTVEYLAAVALDIFVYCYFGNQIILQASRVSTAA YQCAWPAMGVRPRRLLLNILLANKRPVAVRAGRFLPM DLHTFVVIIKTSFSYYTLLDKINN		
PoctOR72	KM892410	1170	MFRETYTIVLKYFRNNLIDVLEEMPWLGRLSWLQFLVY VLAVVSHTGGVLERMGDGADMVQLSGDLSATLVLWQ VTVLYVQIYVNRKLIRNLILNLGSKWSSDDYLSPEMVA VKQQSVKTIYKWITYFYKVLTVFMHLYFCLPLSAAAVK HFVLKEEFAFATIYKLKMPFRYEDNFLLYWIVYMVDYG VLYNTGFLITSDLLLVNVSMNHLRTLFIILQDDLKSIVHS ASEPFERTATRRLKEIIPKHANLLQLMVELSEAFGAIFLI HLAFFSGTMCFFGFAARVHCSPESIKNLLASSFILICIYSC CSCGQYLTDSSLDVANAAYEGSWHLMSHEYRICILFIM LRSQKAYYIKSTSFSDISLQSFTKILNVTWSFLSLITKVYE E	-	-
PoctOrco	KM892411	1425	MMGKVKTQGLVSDLMPNIKLMQAVGHFLFNYTDENG GMSMLLRKIYASTHAVLIVVNFLCMAVNMAQYSDEVN ELTANTITVLFFAHTVIKLLFFAMNSKNFYRTLAVWNQS NSHPLFTESDARYHQLALNKMRRLLYFIGSVTIMAVVS WITITFFGESVRLIADKESNDTLTEPAPRLPLKTWYPFNA MSGTMYIVAFVYQIYWLLFSMAIANLMDVMFCSWLIFA CEQLQHLKAIMKPLMELSASLDTYRPNTSELFRASSTEK SEKVPEPVDMDIRGIYSTQQDFGMLLRGAGGRLQNFNN PNPNNPNGLTQKQEMLARSAIKYWVERHKHVVRLVASI GDTYGTALLFHMLVSTITLTLLAYQATKIDGLNVYAFST VGYLSYTLGQVFHFCIFGNRLIEESSSVMEAAYSCQWY DGSEEAKTFVQIVCQQCQKAMSISGAKFFTVSLDLFASV LGAVVTYFMVLVQLK	-	-

a. - = no detectable sex-biased expression in antennae, male = male-biased expression, female = female-biased expression
